# Eleven new species of theridiosomatid spiders from southern China (Araneae, Theridiosomatidae)

**DOI:** 10.3897/zookeys.255.3272

**Published:** 2012-12-27

**Authors:** Qingyuan Zhao, Shuqiang Li

**Affiliations:** 1 Institute of Zoology, Chinese Academy of Sciences, Beijing 100101, China; 2 University of Chinese Academy of Sciences, Beijing 100049, China

**Keywords:** Biodiversity, taxonomy, canopy, cave, leaf litter

## Abstract

Two new genera of the spider family Theridiosomatidae, *Alaria* **gen.n.** with the type species *Alaria chengguanensis* **sp. n.**, *Menglunia* **gen.n.** with the type species *Menglunia inaffecta* **sp. n.**, are described from Guizhou and Yunnan, China. Nine more new species from Guangxi, Guizhou, Hainan and Yunnan Provinces of southern China are described: *Baalzebub rastrarius* **sp. n.**,*Baalzebub youyiensis* **sp. n.**, *Karstia nitida***sp. n.**, *Karstia prolata* **sp. n.**,*Ogulnius hapalus* **sp. n.**, *Theridiosoma plumaria* **sp. n.**, *Theridiosoma triumphalis* **sp. n.**, *Theridiosoma vimineum* **sp. n.**, *Zoma fascia* **sp. n.** The type specimens are deposited in the Institute of Zoology, Chinese Academy of Sciences in Beijing.

## Introduction

Theridiosomatids are small (usually ≤ 3mm), widely distributed, and cryptozoic spiders, which can be found in damp, dark habitats (litter layer of forest or caves). They can be easily recognized by the presence of a pair of pits on the anterior margin of the sternum near the labial base (Fig. 9D), the disproportionately large globular pedipalps (except for genus *Menglunia*), and the long dorsal trichobothria on the third and fourth tibia (Coddington 1986).

The genera of family Theridiosomatidae were reviewed and revised by Coddington in 1986. Based on cladistic analysis, he recognized 4 subfamilies: Platoninae, Epeirotypinae, Ogulniinae and Theridiosomatinae. He also described/redescribed 9 genera and validated 28 species in his paper. Since then, 5 new genera and 25 new species have been discovered worldwide. In 2011, Wunderlich introduced three new theridiosomatid species from Laos and recognized one new subfamily: Luangnaminae. He also downgraded Platoninae, Epeirotypinae, and Ogulniinae to tribal rank based on rare or special characters. According to Platnick (2012), there are currently 16 genera and 89 species belonging to this family. Three widespread genera including *Ogulnius*, *Theridiosoma*, *Wendilgarda*, can be found in China and other Asian countries. The genus *Coddingtonia* was the first genus known only from China. As a monotypic genus, *Coddingtonia* is distinguished from other theridiosomatids by its distantly-separated spermathecae and long copulatory ducts. Miller et al. (2009) also discovered new species from other six genera, three of which were found in China for the first time. *Zoma dibaiyin* as the second speices and first known male from the genus *Zoma*, gives us new thoughts about the validation of *Theridiosoma taiwanica* (Zhang & Zhu, 2006). According to the illustrations given by that paper, we found that it resembles several critical features possessed by *Zoma* rather than *Theridiosoma*. We have no doubt that it will make more sense if it could be transferred to genus *Zoma*. The genus *Karstia*, including two species, was created by Chen (2010). As the second genus endemic to China, *Karstia* is placed in the Theridiosomatinae for three synapomorphies it shares with the other members of this subfamily: a row of short bristles on the cymbium at the junction with cymbial lamella, elongated median apophysis with a trough or groove along its upper surface, and singly attached egg sacs (Chen 2010: figs 12, 13, 30). Two new species of *Kastia* are described here in this paper, merely on the basis of the female characteristics, further diagnosis will be done when males are collected. To a certain extant, *Karstia* and *Baalzebub* are seemingly closely related, despite the fact *Karstia* has a few unique features: presence of cymbium apophysis, and stout, overlapped spermathecae, the accuracy or verity of it is still questionable to us, and more study efforts desperately need to be put into this extraordinary genus in the future.

Theridiosomatids in China are mainly found in the southern provinces: Guangxi, Guizhou, Hainan, Taiwan and Yunnan, which is consistent with the tropical and subtropical preferences typical for this family. Some theridiosomatids are associated with caves, which are characterized by stable humidity and temperature. In this paper, we provide descriptions and distribution data for eleven new species collected in Guangxi, Guizhou, Hainan and Yunnan.

## Method

Specimens were examined using a LEICA M205 C stereomicroscope. Further details were studied under an Olympus BX51 compound microscope. All illustrations were made using a camera lucida attached to an Olympus BX51 compound microscope, and then inked on ink jet plotter paper. Male and female genitalia were examined and illustrated after being dissected from the spiders’ bodies. Left pedipalps of male spiders were illustrated, except as otherwise indicated. Vulvae of female were removed and cleared in lactic acid or warm 10% potassium hydroxide (KOH) solution before illustration. All embolic divisions and vulvae were illustrated after being embedded in Arabic gum. Type specimens examined were preserved in 75% ethanol solution. Photos were taken with an Olympus c7070 wide zoom digital camera (7.1 megapixels) mounted on an Olympus SZX12 stereomicroscope. Images from multiple focal planes were combined using Helicon Focus (version 3.10.3) image stacking software. All measurements are given in millimeters. Leg measurements are shown as: total length (femur, patella, tibia, metatarsus, tarsus).

SEM images were taken using the HITACHI S-3000N at the Institute of Genetics and Developmental Biology, Chinese Academy of Sciences. Specimens for SEM examination were critical point dried and sputter coated with gold-palladium. Specimens were mounted on copper pedestal using double-sided adhesive tape.

All type specimens are deposited in the Institute of Zoology, Chinese Academy of Sciences in Beijing.

**Chaetotaxy.** Macrosetae are marked for the dorsal (d), prolateral (p), retrolateral (r), and ventral (v) surfaces of the legs. Metatarsal trichobothrium (Tm) is given as the ratio of the distance between the proximal margin of the metatarsus and the root of the trichobothrium divided by the total length of the metatarsus (Locket and Millidge 1953) and Tm value for each leg is given as Tm I, Tm II, Tm III, Tm IV respectively.

**Abbreviations and conventions.** Abbreviations used in the text are given in Table 1. References to figures in cited papers are listed in lowercase type (fig.); Figures of this paper are noted with an initial capital (Fig.).

When extra materials are examined and recorded, and the paratype’s collecting information is the same as holotype’s, it will be implied in brackets as [same data as holotype].

**Table 1.** List of abbreviations used in the text and figures.

**Male pedipalp**	
**Co**	conductor
**E**	embolus
**EA**	embolic apophysis
**MA**	median apophysis
**PC**	paracymbium
**T**	tegulum
**Vulva**	
**CD**	Copulatory duct
**S**	spermatheca
**Spinnerets spigot morphology**	
**AC**	aciniform gland spigot
**AG**	aggregate gland spigot
**ALS**	anterior lateral spinneret
**CY**	cylindrical gland spigot
**FL**	flagelliform gland spigot
**MAP**	major ampullate gland spigot
**mAP**	minor ampullate gland spigot
**PI**	piriform gland spigot
**PLS**	posterior lateral spinneret
**PMS**	posterior median spinneret
**t**	tartipore
**Institution**	
**IZCAS**	Institute of Zoology, Chinese Academy of Sciences

## Taxonomy

### Key to Theridiosomatidae from southern China

**(**Species known from one sex is marked as ‘m’ or ‘f’ to represent their holotype’s sex ‘male’ or ‘female’; if unmarked, it means they are known from both sexes)

**Table:** 

1	Posterior median eyes separated by about their diameter or more (Figs 20C–D)	2
	Posterior median eyes separated by less than 1/2 their diameter or less (Figs 7C–D)	3
2	The turning made by the copulatory ducts bends outwardly (Fig. 20B). Median apophysis mesally with a projection oriented distoventrally (Fig. 19A). Embolic apophysis long, whip-like (Fig. 19C)	*Ogulnius hapalus* sp. n.
–	The turning made by the copulatory ducts bends inwardly. Median apophysis with apex oriented distodorsally. Embolic apophysis filiform (Miller et al. 2009: figs 5C, 3D, 4G)	*Ogulnius barbandrewsi*
3	Females	4
–	Males	21
4	Scape present (Fig. 2B)	5
–	Scape absent (Fig. 16A)	14
5	Scape protruding from beneath the epigynal plate (Fig. 4A)	6
–	Scape protruding from epigyne’s posterior rim (Fig. 7A)	9
6	Scape less sclerotized, smaller and partially exposed (Coddington 1986: fig. 206)	7
–	Scape more sclerotized, and utterly exposed. Spermathecae juxtaposed. Copulatory ducts rise and curl up to form a blunt-tipped projection at each side (Figs 2A, 5D)	*Alaria chengguanensis* sp. n.
7	Spermathecae juxtaposed, unseparated	8
–	Spermathecae separated (Song and Zhu 1994: fig. 5)	(f) *Wendilgarda assmensis*
8	Epigyne with a deeper median invagination of the posterior margin Miller et al. 2009: 11D	*Wendilgarda muji*
–	Epigyne with a blunt tip and concave posterior margin (Zhu and Wang 1992: fig. 3)	*Wendilgarda sinensis*
9	Spermathecae distally fused (Fig. 7B)	10
–	Spermathecae juxtaposed or overlapped (Fig. 11B)	11
10	Scape with a central pit on the epigynal plate. Spermathecae long,narrow, form an arcade-shaped conformation (Figs 7A–B)	*Baalzebub rastrarius* sp. n.
–	Scape with a semi-transparent distal tip. Spermathecae small, elliptical (Figs 9A–B)	(f) *Baalzebub youyiensis* sp. n.
–	Epigyne subtriangular, pointed posteriorly with concave margins so medial part is more acute than lateral part (Miller et al. 2009: fig. 3E)	(f) *Baalzebub nemesis*
11	Spermathecae juxtaposed (Fig. 11B)	12
–	Spermathecae overlapped at the tip (Chen 2010: fig. 6)	13
12	Epigyne small, with a short, distally spherical scape protruding from posterior margin of epigynal plate (Figs 17A, D, E)	(f) *Karstia nitida*sp. n.
–	Epigyne with a long, apiculate scape protruding perpendicularly (slightly tilted) from posterior margin of epigynal plate (Figs 5A, 18E–F)	(f) *Karstia prolata* sp. n.
13	Epigyne with an apiculate, approximately triangular scape. Spermathecae potato-like, bulging and stout (Chen 2010: fig. 20)	*Karstia coddingtoni*
–	Epigyne with a triangular scape protruding from its posterior rim. Spermathecae peanut-shape stout and simple (Chen 2010: fig. 6)	*Karstia upperyangtz*
14	Spermathecae separated by less their diameter or juxtaposed (Fig. 16B)	15
–	Spermathecae separated by nearly their diameter (Miller et al. 2009: fig. 11F)	(f) *Coddingtonia euryopoides*
15	Spermathecae spherical, separated from each other (Fig. 16B)	*Menglunia inaffecta* sp. n.
	Spermathecae juxtaposed (Fig. 2A)	16
16	Abdomen with silver patches forming curved transverse strip (Figs 29C–D)	17
	Abdomen without silver patches forming curved transverse strip	18
17	The upper rim of the spermathecae is the same height as the copulatory ducts (Miller et al. 2009: fig. 11B)	*Zoma didaiyin*
–	The spermathecae is above the loop made by the copulatory ducts (Fig. 29B)	*Zoma fascia* sp. n.
18	Epigyne with a deep atrium, height of opening about one third the width in posterior view (Miller et al. 2009: fig. 3B)	*Epeirotypus dalong*
–	Atrium absent (Miller et al. 2009: fig. 3F) or slit-like (Miller et al. 2009: fig. 13A), height of opening (if visible) much less than one third the width in posterior view	19
19	Epigyne with pair of processes arising from posterolateral margin running toward each other (Miller et al. 2009: fig. 3H)	(f) *Theridiosoma shuangbi*
–	Epigyne without pair of processes arising from posterolateral margin	20
20	Posterior margin of epigyne with median longitudinal slit (Miller et al. 2009: fig. 9A)	*Theridiosoma diwang*
–	Posterior margin of epigyne without median longitudinal slit, but with a median transverse ridge (Zhang et al. 2006: fig. 2)	*Theridiosoma taiwanica*
21	Embolic apophysis absent (Fig. 1C)	22
–	Embolic apophysis present (Fig. 6C)	24
22	Tegulum without tuberculate-textured mesal lobe	23
–	Tegulum with tuberculate-textured mesal lobe. Median apophysis lightly sclerotized, with fine distoventral projection. Conductor a complex of sclerotized and membranous structure enveloping thick embolus for most of its length (Miller et al. 2009: fig. 2A)	*Epeirotypus dalong*
23	Embolus long, whip-like, mostly enveloped in conductor (Figs 1B–D). Median apophysis disproportionately large, orienting and stretching along the longitudinal axis of pedipalp (Figs 1A, 3A)	*Alaria chengguanensis* sp. n.
–	Embolus short, stout (Figs 15B, 17B). Tegulum suboval shaped (Fig. 15D). Median apophysis with a short projection oriented distoventrally (Fig. 15A)	*Menglunia inaffecta* sp. n.
24	Embolic division simple with one filiform embolic apophysis (Fig. 28C)	25
–	Embolic division complex with more than one embolic apophysis (Fig. 24C)	26
25	Exposed embolic apophysis short with a triangular tip, embolus ‘Z’-shaped (Figs 28A–D)	*Zoma fascia* sp. n.
–	Exposed embolic apophysis long; embolic division with a moderate branching (Miller et al. 2009: fig. 10F)	*Zoma dibaiyin*
26	The mesal bristle of the embolic apophysis protruding from beneath the conductor and lying long the mesal side of the conductor itself (Coddington 1986: fig. 198)	27
–	The palp without a mesal bristle of apophysis protruding from beneath the conductor and lying long the mesal side of the conductoritself	28
27	Conductor with a spear-shaped apophysis, above median apophysis (Zhu and Wang 1992: fig. 7)	*Wendilgarda sinensis*
–	Conductor without a spear-shaped apophysis. Palpa tibia with one trichobothrium. Median apophysis sclerotized with concave dorsal margin (Miller et al. 2009: fig. 12E)	*Wendilgarda muji*
28	Embolic apophysis with blunt, spatulate processes, without a mesal bristle (Coddington 1986: fig. 162; Chen 2010: fig. 11)	29
–	Embolic apophysis fragments, filiform, with tips protruding out of conductor (Fig. 24B)	31
29	Median apophysis elongated and has a trough of groove along its upper surface. Cymbium with apophysis (Chen 2010: figs 26–27)	30
–	Median apophysis small, triangular and cleft (Figs 6A, D). Embolic apophysis spatulate arching structure with abruptly acuminated distal ends (Fig. 6C)	*Baalzebub rastrarius* sp. n.
30	Cymbium apophysis small. Paracymbium with a long spine on the distal end (Chen 2009: fig. 13)	*Karstia upperyangze*
–	Cymbium apophysis big, distally crooked. Paracymbium without a long spine (Chen 2009: fig. 27)	*Karstia coddingtoni*
31	Conductor with plumose branching (Fig. 22C). Median apophysis with a curved lobe attenuates distally (Fig. 23A)	(m) *Theridiosoma plumaria* sp. n.
–	Conductor without plumose branching	32
32	Two embolic apophysis fragments form a 'V'-shaped conformation (Figs 24A–B)	(m) *Theridiosoma triumphalis* sp. n.
–	One embolic apophysis fragment forms a beak-shaped conformation (Figs 26A–B). Conductor with a piece of long, pliant apophysis protruding from its ridge and stretching towards median apophysis (Figs 26B, D)	(m) *Theridiosoma vimineum* sp. n.

#### Genus Alaria gen. n.

urn:lsid:zoobank.org:act:A977D6FD-0DFF-448D-A83D-09193762595F

http://species-id.net/wiki/Alaria

##### Type species.

*Alaria chengguanensis* sp. n.

##### Etymology.

The generic epithet is derived from the Latin ‘alarius’, meaning ‘of wings’, which refers to the two projections of the copulatory ducts at each side, which resembles a pair of wings. Gender is feminine.

##### Diagnosis.

**Figure 1.**
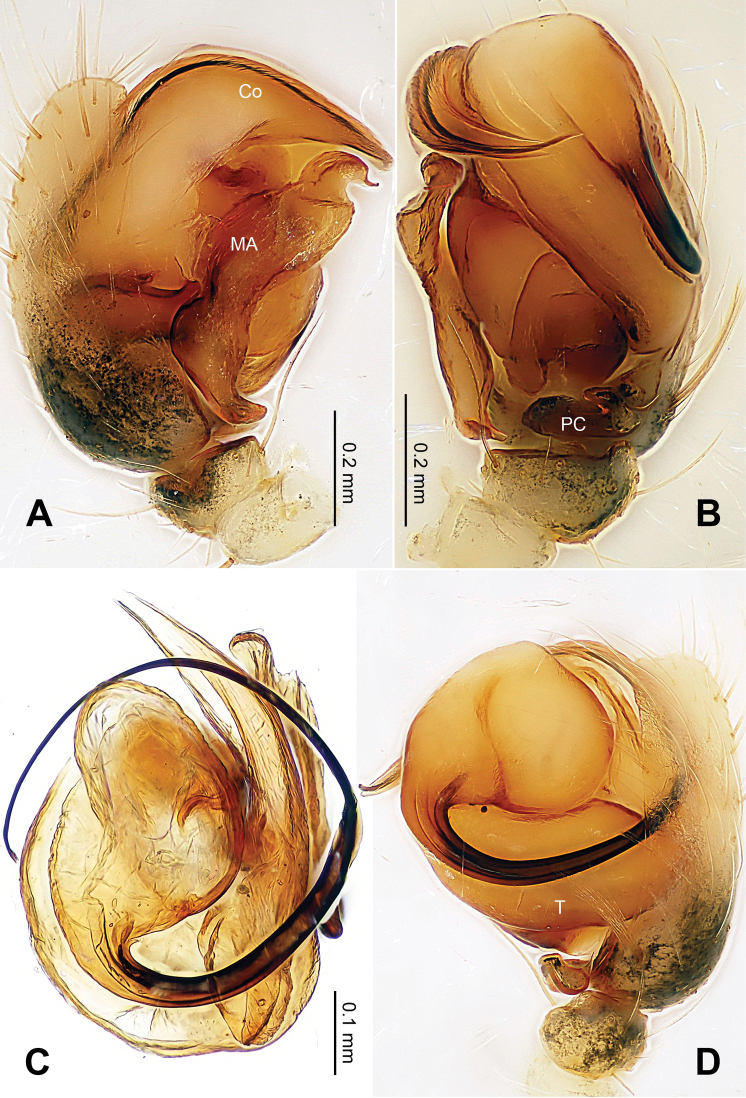
*Alaria chengguanensis* sp. n., male holotype. **A** Pedipalp, prolateral view **B** Pedipalp, ventral view **C** Embolic division, dorsal view **D** Pedipalp, retrolateral view. **Co** conductor; **MA** median apophysis; **PC** paracymbium; **T** tegulum. Scale bars: **D** as **A**.

**Figure 2. F2:**
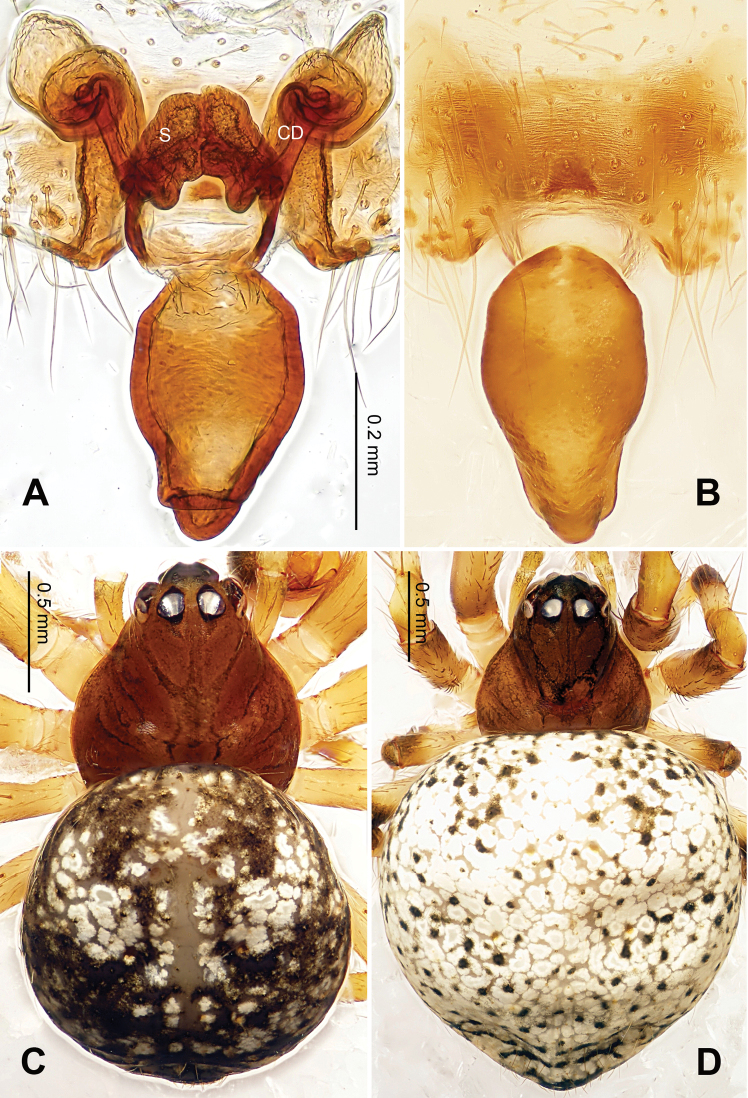
*Alaria chengguanensis* sp. n., male holotype (**C**) and female paratype (**A–B, D**). **A** Vulva, dorsal view **B** Epigyne, ventral view **C** Male, dorsal view **D** Female, dorsal view. **CD** copulatory duct; **S **spermatheca. Scale bars: **B** as **A**.

**Figure 3. F3:**
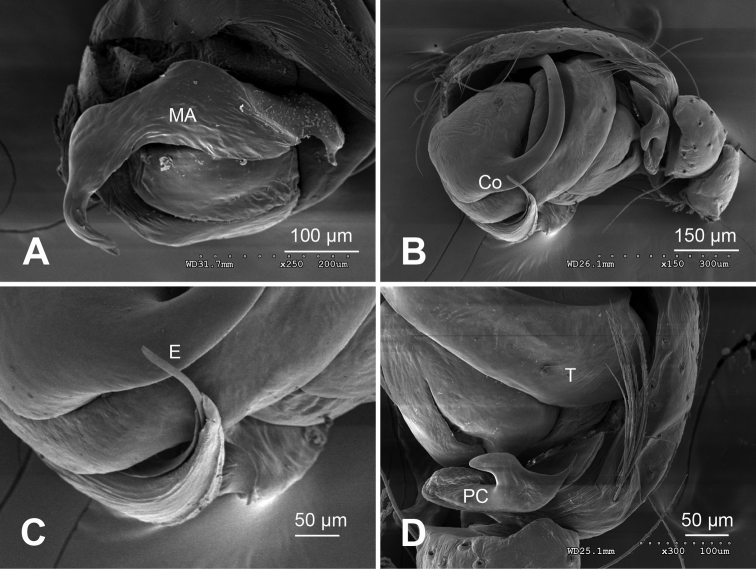
*Alaria chengguanensis* sp. n., SEM of pedipalp of a male paratype. A Prolateral view, detail showing MA **B** Retrolateral view **C** Retrolateral view, detail showingembolus **D** Retrolateral view, detail showing **PC**. **Co** conductor **E** Embolus; **MA** median apophysis; **PC** paracymbium; **T** tegulum.

**Figure 4. F4:**
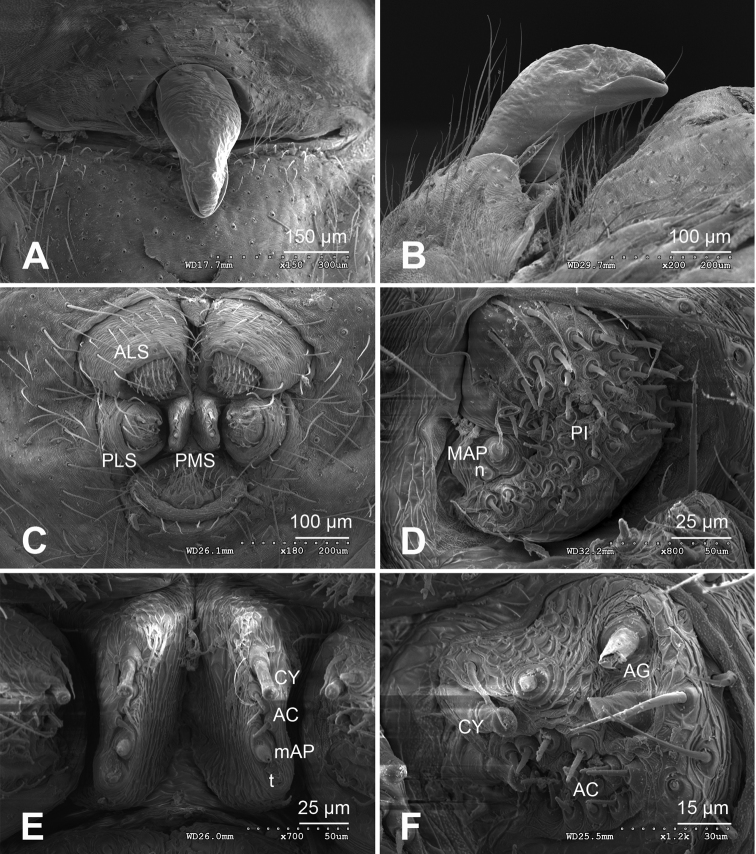
*Alaria chengguanensis* sp. n., SEM of a female paratype. **A** Epigyne, ventral view **B** Epigyne, lateral view **C** Spinnerets **D** ALS **E** PMS **F** PLS. **AC** aciniform gland spigot; **AG** aggregate gland spigot; **ALS** anterior lateral spinneret; **CY** cylindrical gland spigot; **MAP** major ampullate gland spigot; **mAP** minor ampullate gland spigot; **n** nubbin; **PI** piriform gland spigot; **PLS** posterior lateral spinneret; **PMS** posterior median spinneret; **t** tartipore.

The unique structure of epigyne distinguishes *Alaria* from other theridiosomatids. Like in *Wendilgarda* and *Chthonopes*, the scape in female *Alaria* protrudes from beneath epigynal plate (Coddington 1986: figs 206, 213; Wunderlich 2011: figs 18d–e), but is utterly exposed, and more sclerotized, like a shield attached to the abdomen (Figs 2A–B, 4A–B). The conformation of the copulatory ducts is similar to that in *Ogulnius obtectus* (Coddington 1986: fig. 113), but copulatory ducts make one coil before the conjuncture with spermathecae instead a fold (Fig. 2B). The paracymbium in *Alaria* is neither a T-shaped lobe as in most thridiosomatids nor a broad apophysis as in Epeirotypinae, it is a thick, elongated structure with a small hooked projection (Fig. 3D). The long, whip-like embolus in *Alaria* resembles embolic apophysis in *Ogulnius* (Coddington 1986: figs 100–101, 116, 118), but proportionately much longer and mostly enveloped in conductor (Fig. 1B–D). The median apophysis of *Alaria* is disproportionately large, stretching along the longitudinal axis of pedipalp with two curved, pointed distal ends (Figs 1A, 3A), which is never seen in any other theridiosomatid genus. Based on the combination of features mentioned above, *Alaria* should be recognized as a new genus, and is likely close to *Wendilgarda* and *Chthonopes*.

##### Species.

*Alaria chengguanensis* sp. n.

#### Alaria chengguanensis sp. n.

urn:lsid:zoobank.org:act:5EEE5079-3F6D-48CC-84D1-1A26BAD71F78

http://species-id.net/wiki/Alaria_chengguanensis

Figs 1 [Fig F2] [Fig F3] [Fig F4] 5

##### Material examined.

Holotype: CHINA, Guizhou: Bijie City, Chengguan Town, Xiaohe Village, Xiniu Cave, 27°21.231'N, 105°17.186'E, elevation ca 1515 m, 30 April 2007, J. Liu & Y.C. Lin (IZCAS), 1 male.

Paratypes: [same data as holotype] (IZCAS), 13 males, 8 females.

##### Etymology.

This specific name chéng guān ( ) refers to its type locality; adjective.

##### Diagnosis.

See diagnosis for genus.

##### Description.

Carapace brownish red, with symmetric dark veins. Sternum yellow with dark brown margins. Legs yellow, dark brown distally at joints, especially tibiae. Abdomen tan with dark grey and silver patches (Figs 2A–B).

Male pedipalp: Patella with strong sinuous macroseta. Tibia with two trichobothria. Cymbium with small cluster of long setae proximally (Figs 1B, 3D). Paracymbium elongated, curved near base (Figs 1B, 3D). Tegulum smooth. Median apophysis wide, longitudinally grooved, strongly sclerotized, with curved and pointed distal region. Conductor a thick, looped, blade-shaped structure enveloping embolus for most of its length (Figs 1B–D). Embolus long, slim.

**Figure 5.**
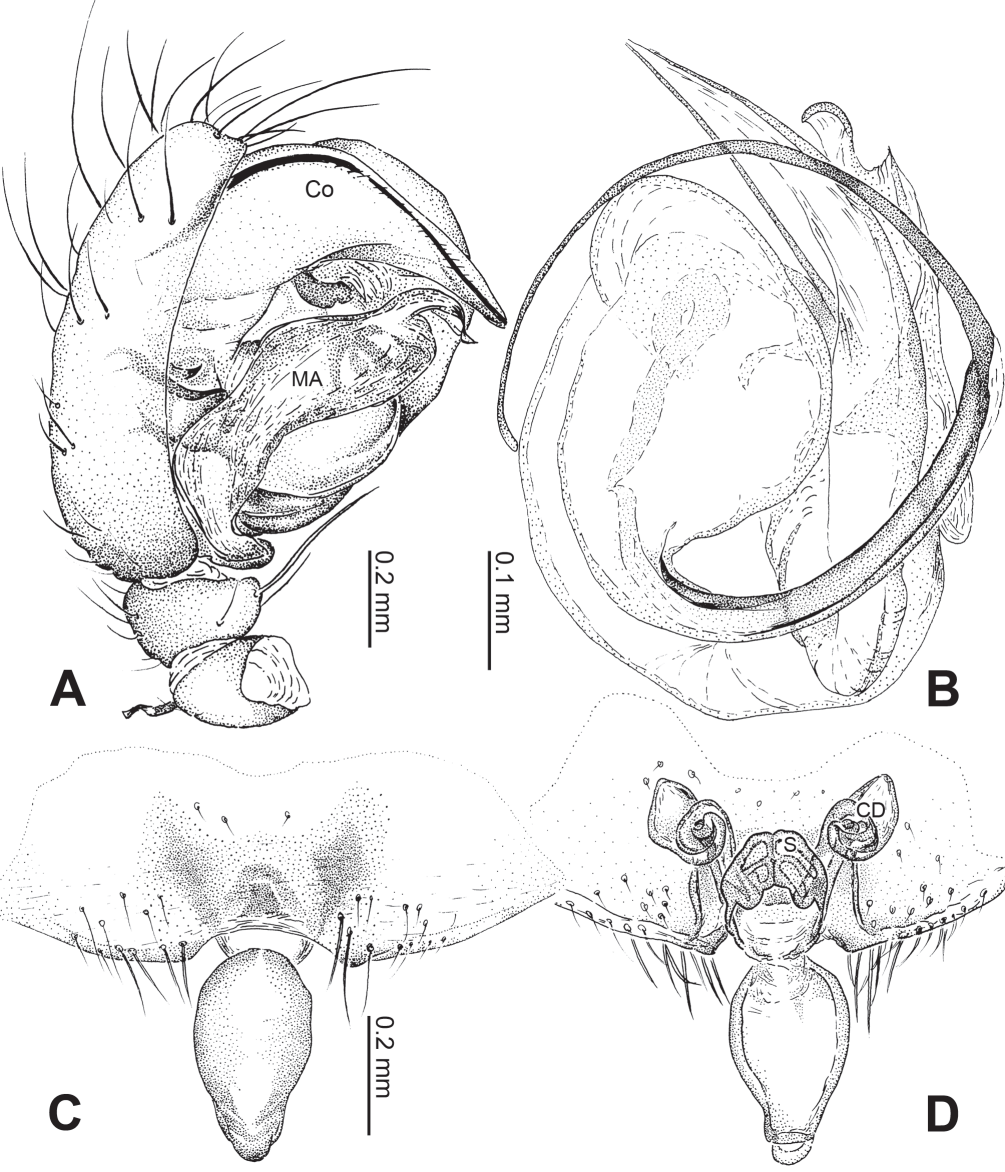
*Alaria chengguanensis* sp. n., male holotype (**A–B**) and female paratype (**C–D**). **A** Pedipalp, prolateral view **B** Embolic division, dorsal view **C** Epigyne, ventral view **D** Vulva, dorsal view. **CD** copulatory duct; **Co** conductor; **MA** median apophysis; **S** spermatheca. Scale bars: **D** as **C**.

Vulva: Epigyne with long, tongue-shaped scape protruding from beneath its concaved margin. Scape slightly humped with a small transverse opening at its distal end. Spermathecae juxtaposed. Copulatory ducts rise and curl up to form a blunt-tipped projection at each side (Figs 2A, 5D).

Male (holotype): Total length 2.10, carapace 1.24 long, 1.08 wide, clypeus 0.13, sternum 0.6 long, 0.6 wide, coxae IV separated by two thirds their width. Posterior median eyes separated by half their diameter. Macrosetae: Leg I: femur d 1, patella d 1, tibia d 1, p 1, v 2, r 1, metatarsus p 1, v 1; Leg II: femur d 2, patella d 2, tibia d 1, p 1, v 1, r 1, metatarsus p 1, v 1; Leg III: femur d 3, patella d 1, tibia p 1, v 2, metatarsus d 1, p 1, r 1; Leg IV: femur d 1, tibia p 1, v 1, r 1, metatarsus p 1, v 1, r 1. Metatarsal trichobothria: Tm I: 0.27; Tm II: 0.30; Tm III: 0.84; Tm IV: 0.24. Leg measurements: I 3.98 (1.25, 0.50, 0.85, 0.88, 0.50); II 3.09 (1.00, 0.43, 0.63, 0.63, 0.40); III 2.27 (0.68, 0.35, 0.38, 0.48, 0.38); IV 2.95 (0.93, 0.38, 0.63, 0.63, 0.38).

Female (one of paratypes): Total length 2.50, carapace 1.25 long, 1.25 wide, clypeus 0.08, sternum 0.78 long, 0.63 wide, coxae IV separated by 1 time their width. Posterior median eyes separated by half their diameter. Macrosetae as in male. Metatarsal trichobothria: Tm I: 0.21 ; Tm II: 0.25; Tm III: 0.48; Tm IV: 0.27. Leg measurements: I 4.66 (1.05, 0.63, 0.95, 0.95, 0.63); II 3.75 (1.20, 0.55, 0.75, 0.75, 0.50); III 2.48(0.75, 0.33, 0.50, 0.50, 0.40); IV 3.75 (1.25, 0.50, 0.75, 0.75, 0.50).

### Genus *Baalzebub* Coddington, 1986

*Baalzebub* Coddington, 1986: 71. Type species *Baalzebub baubo* Coddington, 1986.

#### Baalzebub rastrarius sp. n.

urn:lsid:zoobank.org:act:7E74FF15-FCE5-45FB-807A-12724669D543

http://species-id.net/wiki/Baalzebub_rastrarius

Figs 6 [Fig F7] 8

##### Material examined. 

Holotype:CHINA, Guizhou: Bijie City, Chengguan Town, Xiaohe Village, Xiniu Cave, 27°21.231'N, 105°17.186'E, elevation ca 1515 m, 30 April 2007, J. Liu & Y.C. Lin (IZCAS), 1 male.

Paratypes: [same data as holotype] (IZCAS), 1 male, 7 females. CHINA, Guizhou: Xishui County, Sangmu Town, Tuhe Village, Dongkouwan Cave, 28°15.679'N, 106°18.355'E, elevation ca 1271 m, 16 March 2011, Z.G. Chen & Z.W. Zha (IZCAS), 4 males, 8 females; Dafang County, Wenge Town, Taibai Village, Yelaoda Cave, 27°10.869'N, 105°28.289'E, elevation ca 1398 m, 12 March 2011, Z.G. Chen & Z.W. Zha (IZCAS), 2 males, 2 females.

##### Etymology.

The epithet comes from Latin word ‘rastrarius’ which means ‘of a hoe’, referring to the hoe-shaped conductor in prolateral view; adjective.

##### Diagnosis.

**Figure 6.**
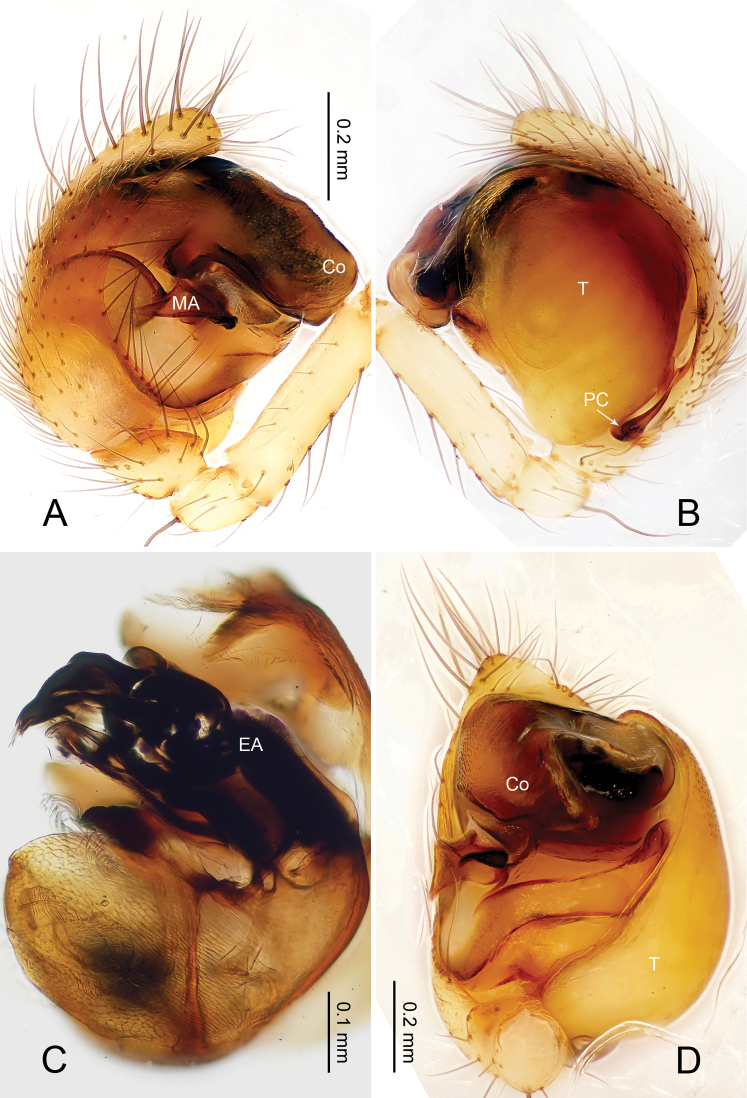
*Baalzebub rastrarius* sp. n., male holotype. **A** Pedipalp, prolateral view **B** Pedipalp, retrolateral view **C** Embolic division, retrolateral view **D** Pedipalp, ventral view. **Co** conductor; **EA** embolic apophysis; **MA** median apophysis; **PC** paracymbium; **T** tegulum. Scale bars: **B** as **A**.

**Figure 7. F7:**
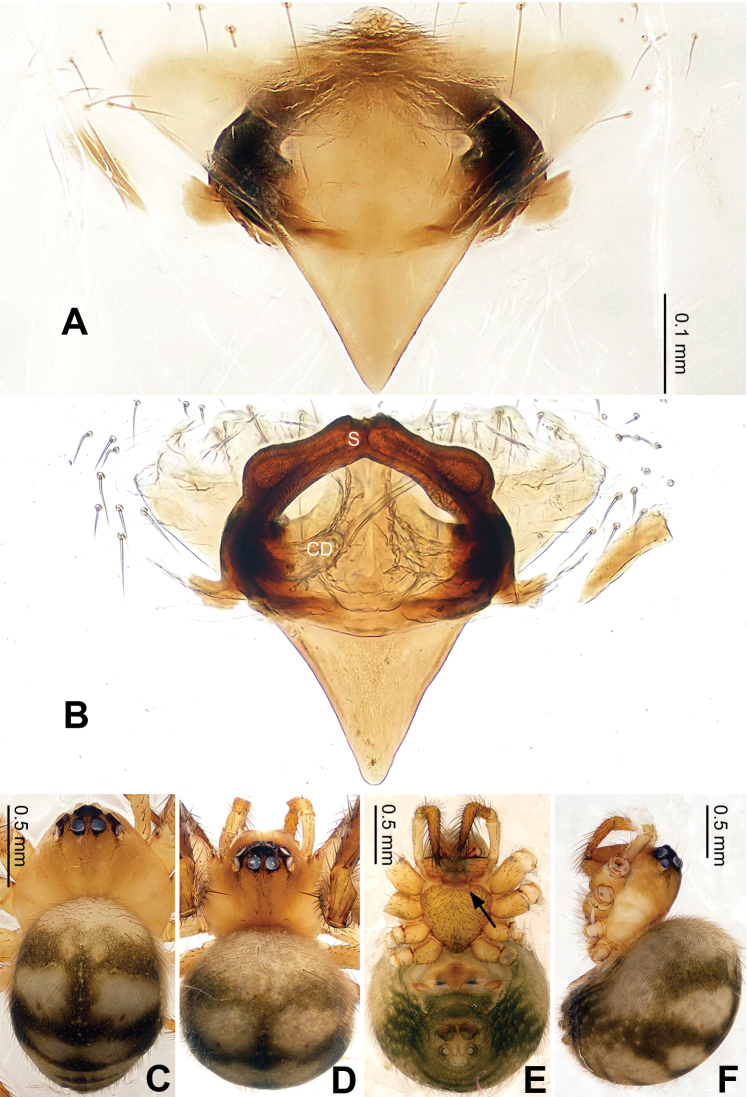
*Baalzebub rastrarius* sp. n., male holotype (**C**) and female paratype (**A–B, D–F**). **A** Epigyne, ventral view **B** Vulva, dorsal view **C** Male, dorsal view **D** Female, dorsal view **E** Female, ventral view **F **Female, lateral view. **CD** copulatory duct, **S** spermatheca. Scale bars: **B** as **A, D** as **E**.

The presence of small, cleft median apophysis and blunt, spatulate processes of embolic apophysis in males, and tip-fused spermathecae in females indicates that this species belongs to the genus *Baalzebub*. Conductor in males envelopes the entire embolic apophysis (Fig 6A), which is similar to *Baalzebub albinotatus*, butthe rectangular conductor and the small, pointed cymbium apophysis are different from other described *Baalzebub* species. Females distinguished by the triangular epigynal plate, similar to *Baalzebub baubo* (Coddington 1986: figs 183, 184), but distinguished by the narrower, arcade-shaped spermathecae (Fig. 7B).

##### Description.

Carapace yellow tan. Sternum yellow with dark orange margins and setae. Legs yellow, dark brown distally at joints, especially tibiae. Abdomen tan with 2–4 rows of dark grey patches, the first two of which are connected by a perpendicular grey patch at midline, and two pairs of small dorsal brown spots (Figs 7C–F).

**Figure 8.**
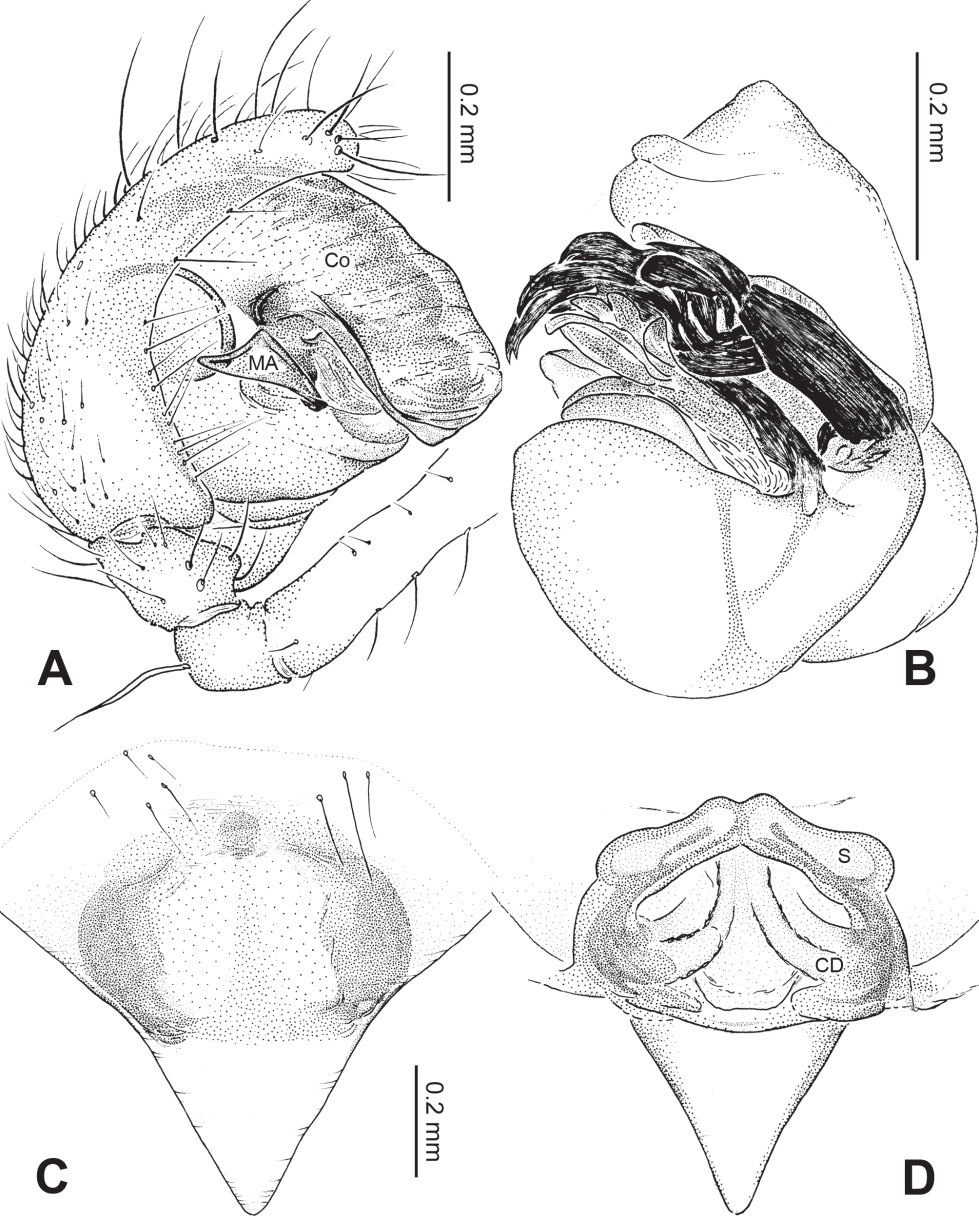
*Baalzebub rastrarius* sp. n., male holotype (**A–B**) and female paratype (**C–D**). **A** Pedipalp, prolateral view **B** Embolic division, retrolateral view **C** Epigyne, ventral view **D** Vulva, dorsal view. **CD** copulatory duct; **Co** conductor; **MA** median apophysis; **S** spermatheca. Scale bars: **D** as **C**.

Male pedipalp: Patella with strong sinuous macroseta. Tibia with two trichobothria. Cymbium with a row of short regular bristles at its junction with cymbial lamella. Paracymbium with a long, pointed distal end (Fig. 6B). Cymbium apophysis small and pointed (Fig. 6B). Median apophysis triangular, strongly sclerotized (Figs 6A, 8A). Conductor a sub-rectangular translucent theca covering the complex embolic division and embolic apophysis. Embolic apophysis a spatulate arching structure with abruptly acuminated distal ends (Fig. 6C).

Vulva: Epigyne with a smooth triangular scape protruding from posterior margin of epigyne plate (Figs 7A–B). A deep pit lies on the epigynal mesal anterior edge. Spermathecae long, narrow, meeting at the tip to form an arcade-shaped structure. Copulatory ducts follow simple curve (Figs 7B, 8D).

Male: Total length 1.75, carapace 0.94 long, 1.09 wide, clypeus 0.25, sternum 0.53 long, 0.56 wide, coxae IV separated by their width. Posterior median eyes separated by less than half their diameter. Macrosetae: Leg I: femur p 1, patella d 1, tibia d 3, r 1; Leg II: femur r 1, patella d 2, tibia d 1, r 1; Leg III: patella d 2, tibia d 1; Leg IV: patella d 2, tibia d 1. Metatarsal trichobothria: Tm I: 0.25; Tm II: 0.29; Tm III: 0.23; Tm IV: 0.26. Leg measurements: I 4.66 (1.50, 0.50, 1.26, 1.00, 0.40); II 3.45 (1.10, 0.40, 0.80, 0.80, 0.35); III 2.50 (0.75, 0.30, 0.50, 0.50, 0.45); IV 3.10 (0.75, 0.60, 0.75, 0.70, 0.30).

Female: Total length 2.24, carapace 0.93 long, 0.93 wide, clypeus 0.11, sternum 0.5 long, 0.5 wide, coxae IV separated by their width. Posterior median eyes separated by less than half their diameter. Macrosetae as in male. Metatarsal trichobothria: Tm I: 0.28; Tm II: 0.25; Tm III: 0.45; Tm IV: 0.27. Leg measurements: I 4.17 (1.38, 0.48, 0.98, 0.88, 0.45); II 3.39 (1.10, 0.43, 0.73, 0.75, 0.38); III 2.50 (0.75, 0.35, 0.50, 0.55, 0.35); IV 3.11 (1.00, 0.33, 0.65, 0.75, 0.38).

#### Baalzebub youyiensis sp. n.

urn:lsid:zoobank.org:act:791FC4A5-1C1F-4E54-97BA-EEDF7FAFFCA9

http://species-id.net/wiki/Baalzebub_youyiensis

Figs 9 10

##### Material examined.

Holotype: CHINA, Guangxi: Pingxiang City, Youyi County, Bantou Village, Niuyan Cave, 22°05.666'N, 106°45.439'E, elevation ca 251 m, 18 January 2011, Z.G. Chen & Z.W. Zha (IZCAS), 1 female.

Paratypes: [same data as holotype] (IZCAS), 3 females.

##### Etymology.

This specific name formed from the Chinese words for friendship yŏu yì ( ), which is the name of the county where this species was collected; adjective.

##### Diagnosis.

**Figure 9.**
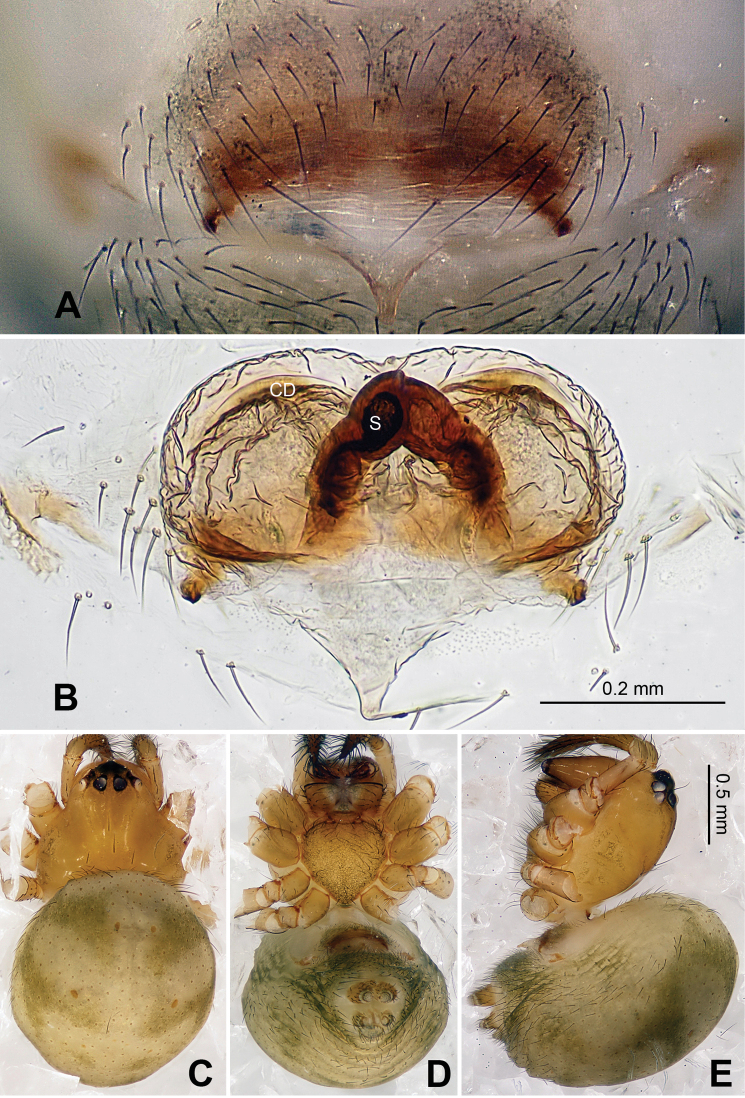
*Baalzebub youyiensis* sp. n., female holotype. **A** Epigyne, ventral view **B** Vulva, dorsal view **C **Female, dorsal view **D** Female, ventral view **E** Female, lateral view. **CD** copulatory duct; **S** spermatheca. Scale bars: **A** as **B, C, D** as **E**.

Females distinguished from other described *Baalzebub* by the shape of the semi-transparent scape, which is proportionately shorter and with a blunt tip (Fig. 9A). Spermathecae, contrasted with narrow, long spermathecae in other *Baalzebub* species, is relatively shorter and smaller compared to the ovoid loops made by copulatory ducts (Fig. 9B)

##### Description.

Carapace broad, orange. Sternum yellow with dusky margins. Legs yellow, brown from patella to tarsus (except for leg I, which only brown distally at joints). Abdomen beige with irregular light, greenish-grey patches (Figs 9C–E).

**Figure 10.**
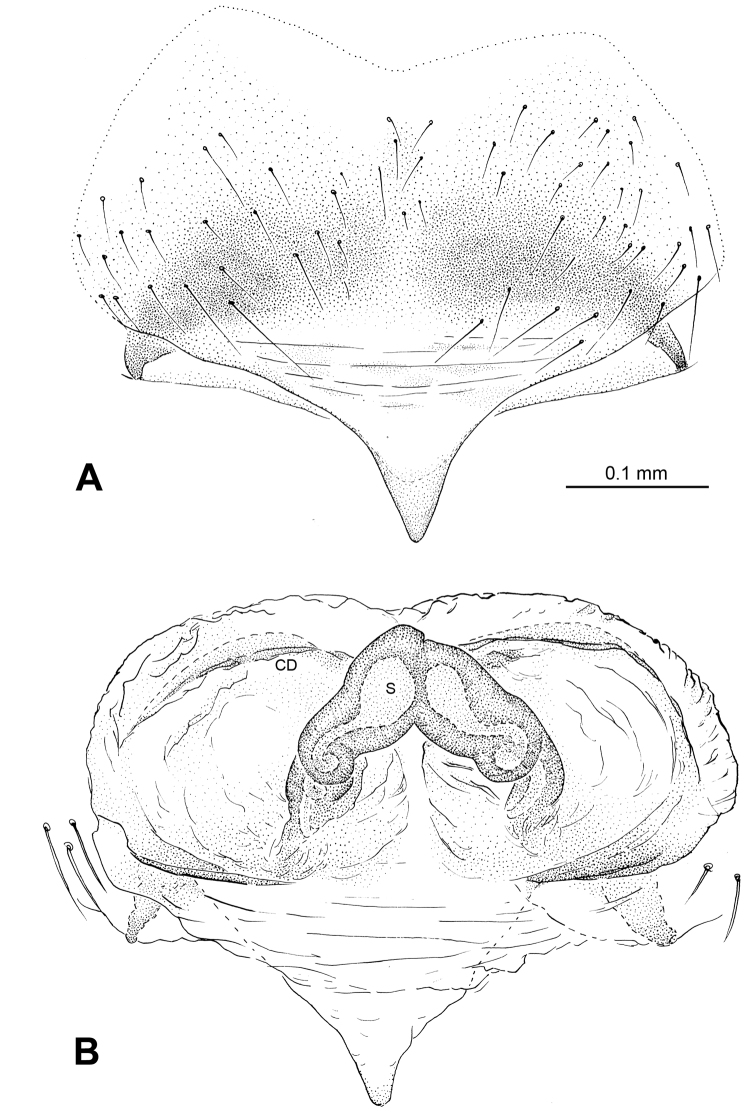
*Baalzebub youyiensis* sp. n., female holotype. **A** Epigyne, ventral view **B** Vulva, dorsal view. **CD** copulatory duct; **S** spermatheca. Scale bars: **B** as **A**.

Vulva: Epigyne with a short, pointed triangular scape with concave lateral margins protruding from posterior margin of epigyne plate, through which dark orange vulva is visible (Fig. 9A). Scape translucent. Epigyne plate with transverse grooves (Fig. 9A). Spermathecae small, elliptical, joining each other at the tip (Figs 9B, 10B). Copulatory ducts simple, with three coils toward spermathecae.

Female: Total length 2.10, carapace 1.00 long, 1.00 wide, clypeus 0.19, sternum 0.53 long, 0.53 wide, coxae IV separated by 1.00 time their width. Posterior median eyes separated by less than half their diameter. Macrosetae: Leg I: femur d 1, p 1, patella d 1, tibia d 2, p 1, r 1; Leg II: patella d 3, tibia d 2, r 1; Leg III: patella d 2, tibia d 2, r 2; Leg IV: patella d 2, tibia d 1. Metatarsal trichobothria: Tm I: 0.20; Tm II: 0.15; Tm III: 0.19; Tm IV: 0.22. Leg measurements: I 3.76 (1.20, 0.40, 0.88, 0.80, 0.48); II 3.2 (1.00, 0.36, 0.72, 0.64, 0.48); III 2.24 (0.72, 0.32, 0.44, 0.45, 0.31); IV 2.92 (1.00, 0.40, 0.60, 0.52, 0.40).

Male unknown.

### Genus *Karstia* Chen, 2010

*Karstia* Chen, 2010: 2. Type species *Karstia upperyangtzica* Chen, 2010.

#### Karstia nitida sp. n.

urn:lsid:zoobank.org:act:5ABD3E69-462D-4811-9851-EF178144FE62

http://species-id.net/wiki/Karstia_nitida

Figs 11 12

##### Material examined.

Holotype: CHINA, Guangxi: Hechi City, Hechi County, Laba Village, Shoushui Cave, 24°41.229'N, 107°52.609'E, elevation ca 268 m, 31 March 2011, Z.G. Chen & Z.W. Zha (IZCAS), 1 female.

Paratypes: [same data as holotype] (IZCAS), 14 females.

##### Etymology.

This specific name comes from the Latin word ‘nitidus’ which means ‘shinning and elegant’, referring to the glossiness of the swollen tip of the epigynal scape; adjective.

##### Diagnosis.

**Figure 11.**
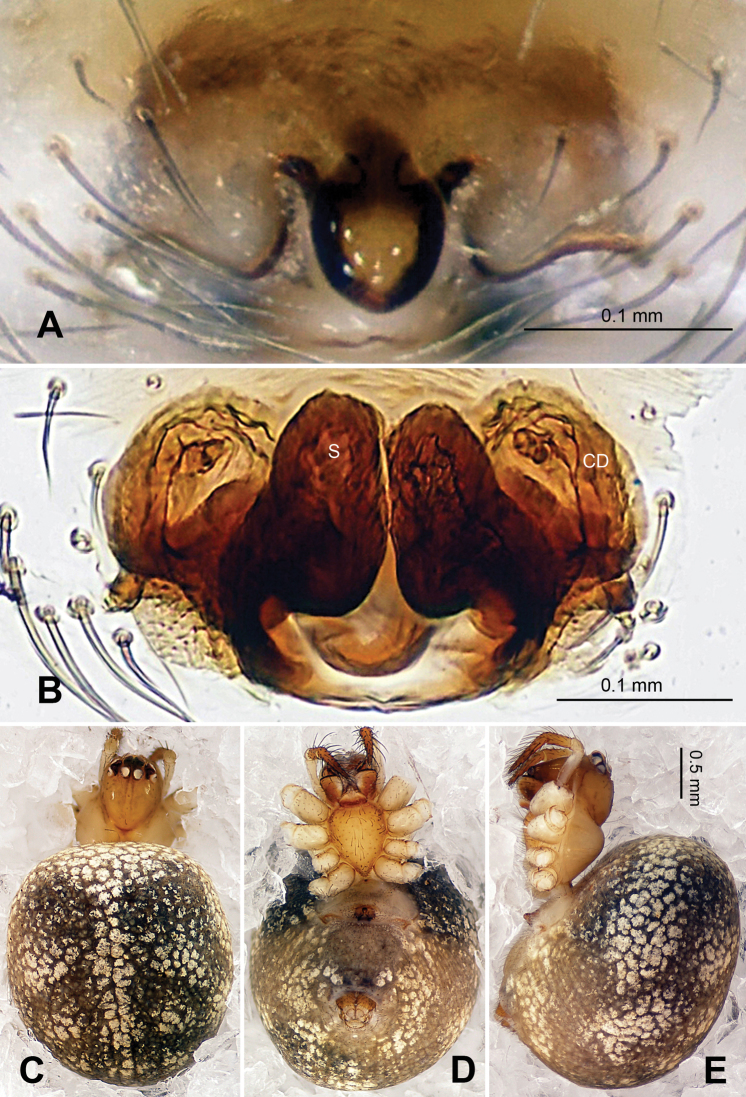
*Karstia nitida* sp. n., female holotype. **A** Epigyne, ventral view **B** Vulva, dorsal view **C** Habitus, dorsal view **D** Habitus, ventral view **E** Habitus, lateral view. **CD** copulatory duct **S** spermatheca. Scale bars: **C, D** as **E**.

Females distinguished by the following combination of characters: the structure of the scape, the stout, overlapped spermathecae (Fig. 11B), and the habitus of this species (Figs 11C–E). Spermathecae oval-shaped with vertically longer diameter, slightly detached from each other along their inner margin. The abdomen large, contrasted with distinctly small epigynal area. The scape structure is quite different from other *Karstia* or *Baalzebub* species: the tip of scape is swollen and shimmery, and the lateral margins of the plate extend toward the scape tip to form a armet-shaped conformation (Fig. 11A). Generic placement tentative pending discovery and examination of the male.

##### Description.

Carapace pale yellow with yellow ocular region. Sternum yellow with tan margins. Legs yellow, brown distally at joints. Abdomen dark grey mottled with white patches (Figs 11C–E).

**Figure 12.**
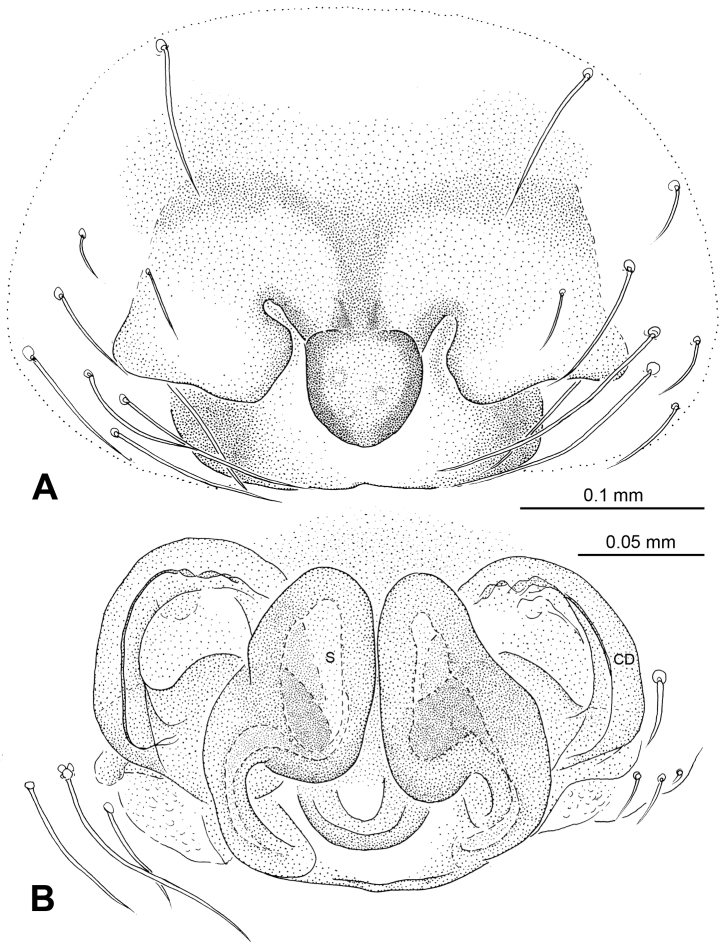
*Karstia nitida* sp. n., female holotype. **A** Epigyne, ventral view **B** Vulva, dorsal view. **CD** copulatory duct, **S** spermatheca.

Vulva: Epigyne small, with short, distally spherical scape protruding from posterior margin of epigyne plate. Epigyne plate extends posteriorly, together with scape to form a barbute-shaped conformation (Figs 11A, 12A). End of scape glossy with black purfle (Fig. 11A). Spermathecae peanut-shaped, juxtaposed, slightly detached from each other (Figs 11B, 12B). Copulatory ducts follow simple rout to form small loops and one turning before connected with spermathecae at the bottom (Figs 11B, 12B).

Female: Total length 3.25, carapace 1.38 long, 1.13 wide, clypeus 0.10, sternum 0.70 long, 0.65 wide, coxae IV separated by their width. Posterior median eyes separated by less than half their diameter. Macrosetae: Leg I: femur p 1, r 1, patella d 1, tibia d 2, p 2, v 2, r 1, metatarsus d 1, v 2, r 1; Leg II: femur d 2, patella d 2, tibia d 4, p 1, v2, metatarsus p 1, v 2; Leg III: femur d 1, patella d 2, tibia d 1, v 1, metatarsus d 3; Leg IV: patella d 2, tibia d 1, v 1, r 1, metatarsus d 2. Metatarsal trichobothria: Tm I: 0.23; Tm II: 0.21; Tm III: 0.16; Tm IV 0.22. Leg measurements: I 4.00 (0.56, 0.63, 1.09, 1.09, 0.63); II 4.16 (1.25, 0.50, 0.94, 0.94, 0.53); III 2.98 (0.78, 0.47, 0.63, 0.63, 0.47); IV 3.66 (1.25, 0.38, 0.78, 0.78, 0.47).

Male unknown.

#### Karstia prolata sp. n.

urn:lsid:zoobank.org:act:38E8200D-E27D-4710-ABEE-FBF657944027

http://species-id.net/wiki/Karstia_prolata

Figs 13 14

##### Material examined.

Holotype: CHINA, Guangxi: Pingxiang City, Youyi County, Bantou Village, Niuyan Cave, 22°05.666'N, 106°45.439'E, elevation ca 251 m, 18 January 2011, Z.G. Chen & Z.W. Zha (IZCAS), 1 female.

Paratypes: [same data as holotype] (IZCAS), 9 females.

##### Etymology.

The specific name is derived from the Latin word ‘prolatus’ meaning elongated’, and refers to the extended epigynal scape; adjective.

##### Diagnosis.

**Figure 13.**
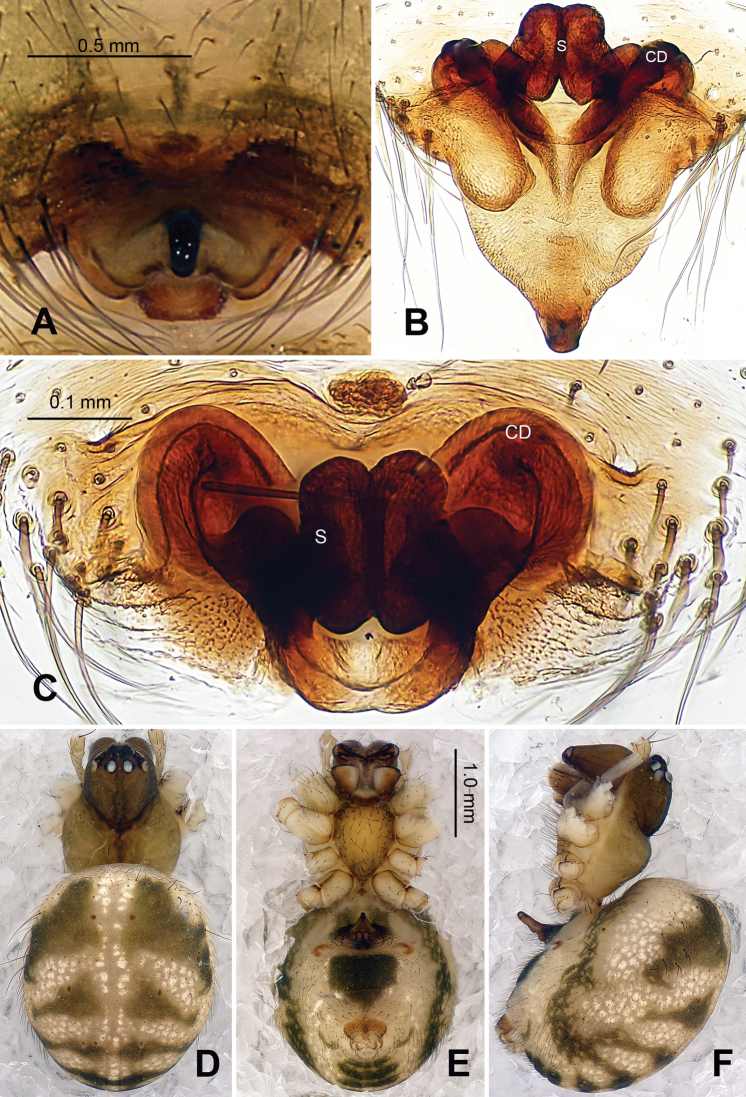
*Karstia prolata* sp. n., female holotype. **A** Epigyne, ventral view **B** Vulva, posterior view **C** Vulva, dorsal view **D** Habitus, dorsal view **E** Habitus, ventral view **F** Habitus, lateral view. **CD** copulatory duct; **S** spermatheca. Scale bars: **B** as **A, D, F** as **E**.

Females distinguished by the protruding scape and the overlapped, stout spermathecae. The long acute-angled scape protrudes vertically from the posterior epigynal margin (Figs 13B, F), which is different from other known *Karstia* species and *Baalzebub* species.

##### Description.

Relatively large in total body length, compared to other theridiosomatid species. Carapace greenish tan, with brown ocular region. Sternum yellow with dark margins. Femur yellow, brown from patella to tarsus. Abdomen beige with evenly distributed silver spots within dorsal area and 8 or more rows of mesally disrupted greenish grey patches, with a large, wedge-shaped greenish grey patch between epigyne and spinnerets (Figs 13D–E).

**Figure 14.**
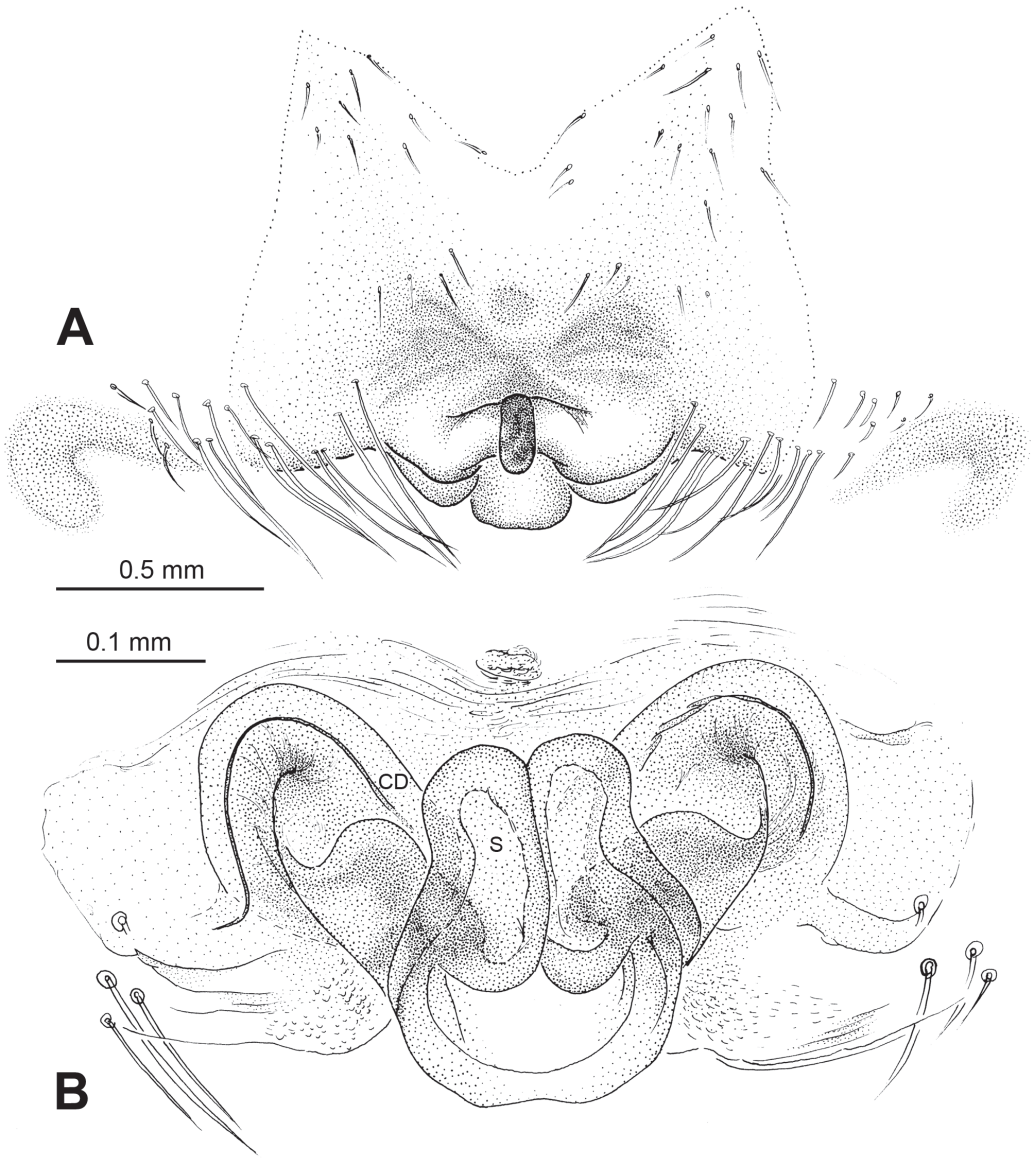
*Karstia prolata* sp. n., female holotype. **A** Epigyne, ventral view **B** Vulva, dorsal view. **CD** copulatory duct; **S** spermatheca.

Vulva: Epigyne with a long, apiculate scape protruding perpendicularly (slightly tilted) from posterior margin of epigyne plate, flanked by a cluster of long sinuous setae on each side (Figs 13A, E–F). Two deep grooves occur at the posterior base of epigyne which are likely connected to the copulatory openings (Figs 13B, E). Tip of scape glossy black (Fig. 13A). Spermathecae peanut-shaped, juxtaposed (Fig. 13C). Copulatory ducts’ routing simple, half-looped (Figs 13C, 14B).

Female: Total length 3.85, carapace 1.92 long, 1.25 wide, clypeus 0.15, sternum 0.63 long, 0.65 wide, coxae IV separated by 2.00 times their width. Posterior median eyes separated by half their diameter. Macrosetae: Leg I: femur d 2, p 1, patella d 2, tibia d 2, p 2, v 1, r 1, metatarsus p 1, v 3, r 1; Leg II: femur d 2, patella d 2, tibia d 1, p 2, v 1, r 1, metatarsus p 1, v 2, r 1; Leg III: femur d 1, v 1, patella d 1, tibia d 1, p 2, v 2, metatarsus d 3; Leg IV: tibia d 2, p 1, metatarsus d 2. Metatarsal trichobothria: Tm I: 0.13; Tm II: 0.22; Tm III: 0.27; Tm IV: 0.22. Leg measurements: I 6.35 (2.00, 0.75, 1.35, 1.50, 0.75); II 5.25 (1.50, 0.70, 1.10, 1.20, 0.75); III 3.35 (0.95, 0.45, 0.70, 0.75, 0.50); IV 4.60 (1.50, 0.50, 1.00, 1.00, 0.60).

Male unknown.

#### Genus Menglunia gen. n.

urn:lsid:zoobank.org:act:272F2972-D45B-4401-AD1E-62ADCB6D498E

http://species-id.net/wiki/Menglunia

##### Type species.

*Menglunia inaffecta* sp. n.

##### Etymology.

The generic epithet refers to the place měng lún ( ) where these specimens were collected. Menglun Town is located at Xishuangbanna in Yunnan province, where tropical rain forest harbors countless species, both vertebrates and invertebrates. Gender is feminine.

##### Diagnosis.

**Figure 15.**
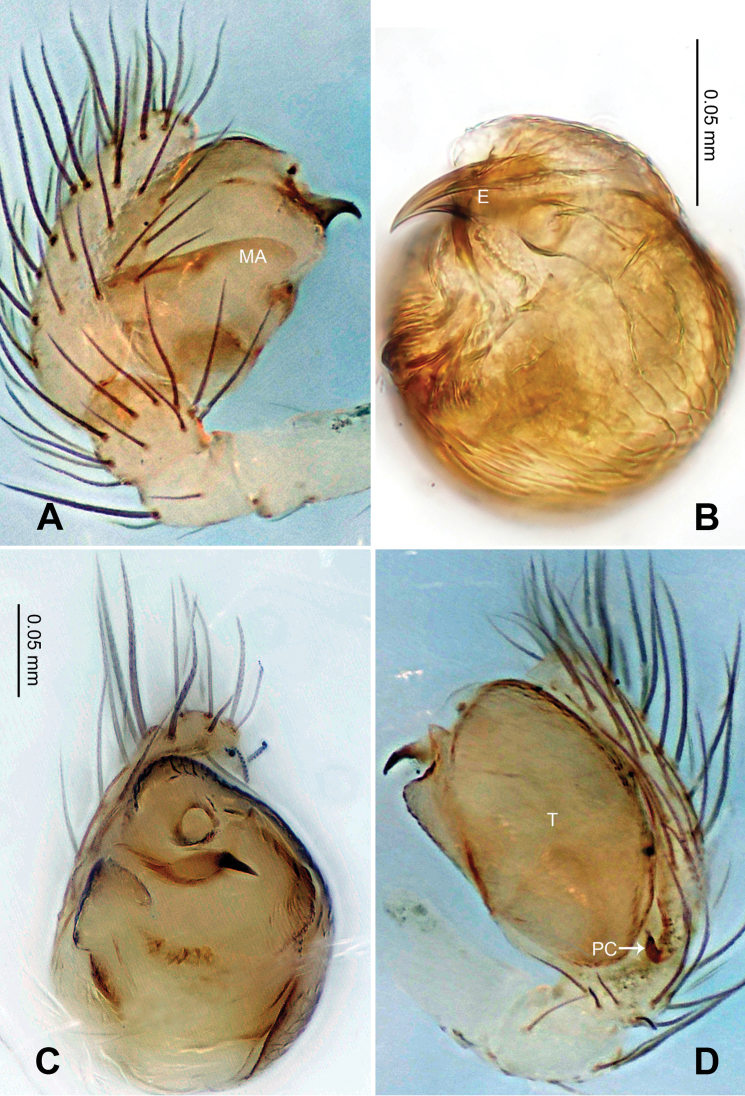
*Menglunia inaffecta* sp. n., male holotype. **A** Pedipalp, prolateral view **B** Embolic division **C** Pedipalp, ventral view **D** Pedipalp, retrolateral view. **E** embolus; **MA** median apophysis; **PC** paracymbium; **T** tegulum. Scale bars: **A, D** as **C**.

**Figure 16. F16:**
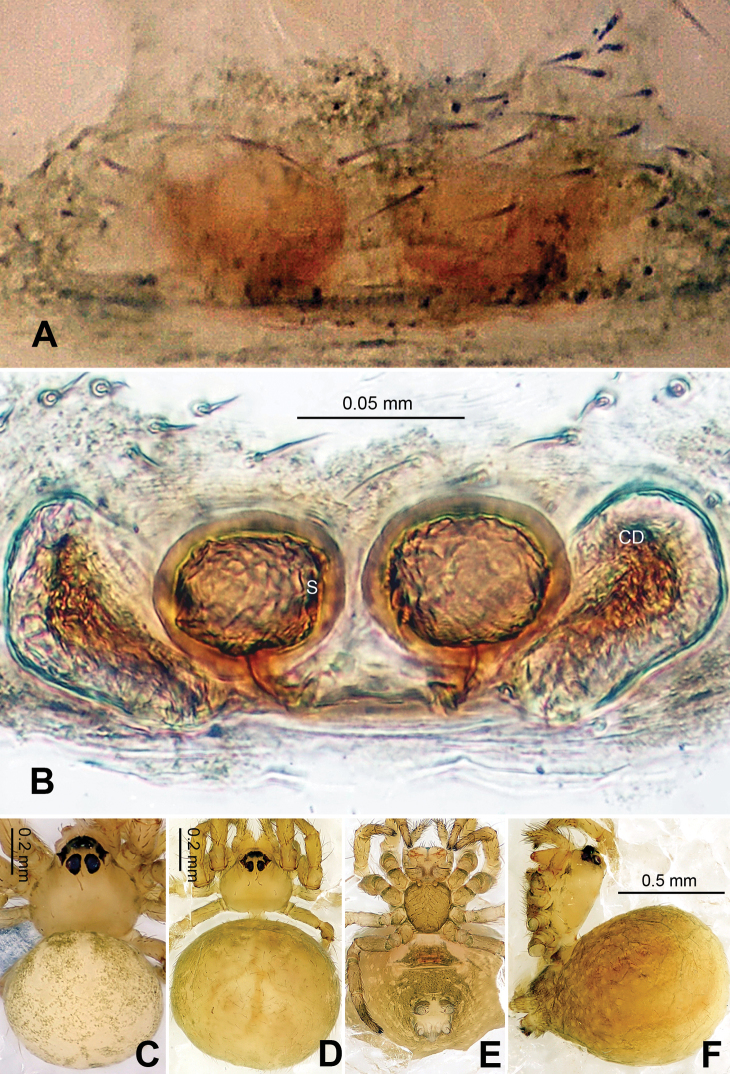
*Menglunia inaffecta* sp. n., male holotype (**C**) and female paratype (**A–B, D–F**). **A** Epigyne, ventral view **B** Vulva, dorsal view **C** Male habitus, dorsal view **D** Female habitus , dorsal view **E **Female habitus, ventral view **F** Female habitus, lateral view. **CD** copulatory duct, **S** spermatheca. Scale bars: **A** as **B, E** as **D**.

Distinguished from other theridiosomatids by the extremely simple, short embolus, and the round, separated spermathecae. The pedipalp in males is an elliptical (slightly rectangular in total), theca-textured, and obscurely circumscribed structure (Figs 15A, D). Conductor is less extensive, and fully covers the embolus (Fig. 18A). The embolus is beak-like, and enveloped in conductor (Fig. 15B). Unlike any other theridiosomatid genus, the median apophysis in *Menglunia* is merely a small projection, mildly curved without any sharp tip or trough (Fig. 15A). Spermathecae similar to *Coddingtonia euryopoides* (Miller et al. 2009) and *Luangnam discobulbus* (Wunderlich, 2011), but instead of being elliptical and separated by their diameter, they are more rounded and separated by less than half of their diameter. Copulatory duct is shorter and forms a simple loop which is about the same height as spermathecae’s diameter, compared to the big loop and higher-positioned copulatory ducts in *Coddingtonia euryopoides and L. discobulbus* (Fig. 16B).

##### Species.

*Menglunia inaffecta* sp. n.

#### Menglunia inaffecta sp. n.

urn:lsid:zoobank.org:act:63DFDE88-8154-412E-B02E-47A71157F286

http://species-id.net/wiki/Menglunia_inaffecta

Figs 15 [Fig F16] [Fig F17] 18

##### Material examined.

Holotype: CHINA, Yunnan: Menglun Town: Xishuangbanna Botanical Garden, 21°55.035'N, 101°16.500'E, elevation ca 558 m, 22 July 2007, primary tropical seasonal rain forest, searching, G. Zheng (IZCAS), 1 male.

Paratypes: [same data as holotype] (IZCAS), 9 males, 9 females.

##### Etymology.

Its Latin origin ‘inaffectus’ means ‘natural and simple’, which refers to the simplicity of the structure of the male pedipalp; adjective.

##### Diagnosis.

See diagnosis for genus.

##### Description.

Carapace yellow tan. Sternum yellow with greenish brown margins and sparse hairs. Legs thick, yellow. Abdomen beige with small, irregularly-distributed greenish brown spots.

**Figure 17. F17:**
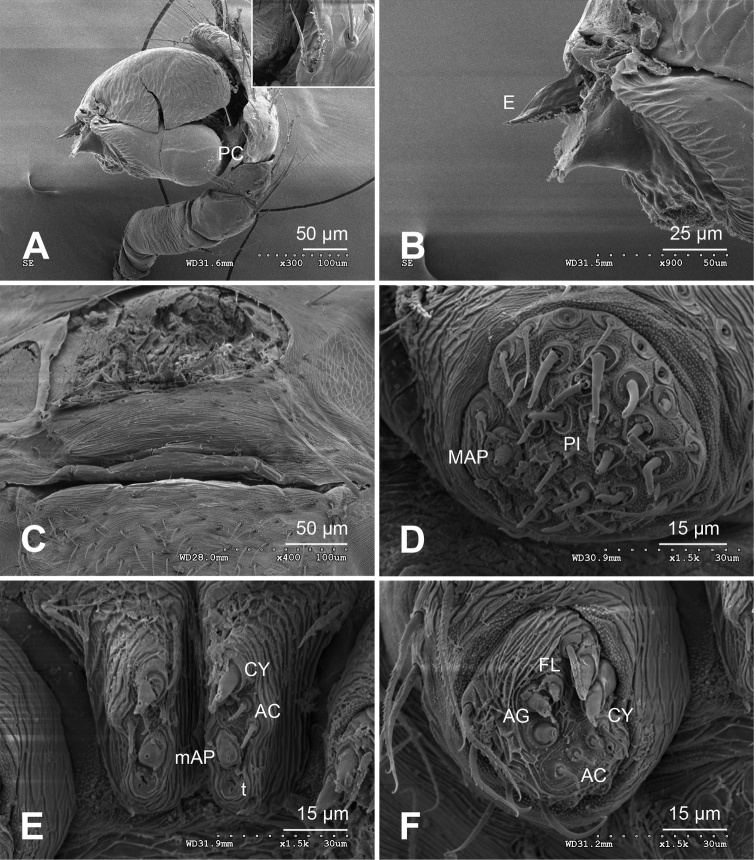
*Alaria chengguanensis* sp. n., SEM of male paratype (**A–B**) and female paratype (**C–F**). **A** Pedipalp, retrolateral view **B** Pedipalp, retrolateral view, detail showing embolus **C** Epigyne **D** ALS **E **PMS **F** PLS. **AC** aciniform gland spigot; **AG** aggregate gland spigot; **ALS** anterior lateral spinneret; **CY** cylindrical gland spigot **E** embolus; **FL** flagelliform gland spigot; **MAP** major ampullate gland spigot; **mAP** minor ampullate gland spigot; **PC** paracymbium; **PI** piriform gland spigot; **PLS** posterior lateral spinneret; **PMS** posterior median spinneret; **t** tartipore.

**Figure 18.**
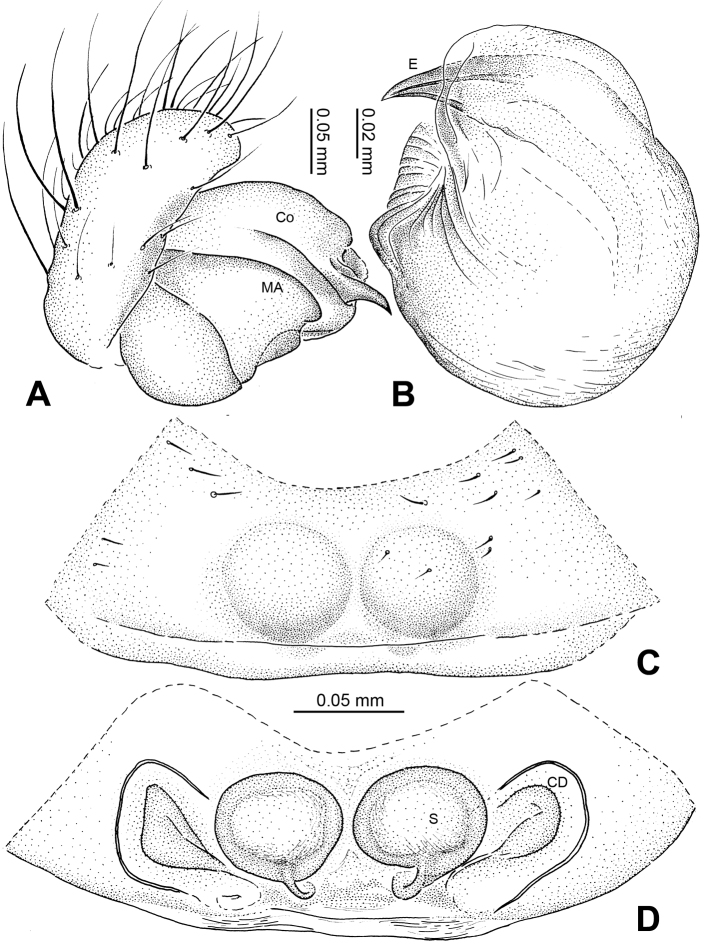
*Menglunia inaffecta* sp. n., male holotype (**A–B**) and female paratype (**C–D**). **A** Pedipalp, prolateral view **B** Embolic division, retrolateral **C** Epigyne, ventral view **D** Vulva, dorsal view. **CD** copulatory duct; **Co** conductor; **MA** median apophysis **S** spermatheca.

Male pedipalp: Patella with erect macroseta. Tibia with one trichobothrium. Paracymbium with a short pointed distal end (Fig. 17A). Tegulum sub-oval. Median apophysis with a small projection oriented distoventrally (Figs 15A, 18A). Conductor kidney-shaped, translucent theca. Embolus beak-shaped, stout (Figs 15A–B, 17B).

Vulva: Epigyne with obscure plate margins (Figs 16A, 17A). Spermathecae round, separated from each other (Fig. 16B). Copulatory duct short and simple (Fig. 16B).

Male (holotype): Total length 1.00, carapace 0.40 long, 0.44 wide, clypeus 0.05, sternum 0.30 long, 0.25 wide, coxae IV separated by 1.5 times their width. Posterior median eyes separated by less than half their diameter. Macrosetae: Leg I: patella d 2, tibia d 2, p 1; Leg II: patella d 1, tibia d 1, p 1, r 1; Leg III: patella d 2. Metatarsal trichobothria: Tm I: 0.27; Tm II: 0.38; Tm III: 0.36. Leg measurements: I 1.10 (0.31, 0.13, 0.25, 0.23, 0.18); II 0.84 (0.25, 0.06, 0.20, 0.20, 0.13); III 0.66 (0.18, 0.11, 0.13, 0.13, 0.11); IV 0.76 (0.19, 0.13, 0.19, 0.14, 0.11).

Female (one of paratypes): Total length 1.00, carapace 0.63 long, 0.48 wide, clypeus 0.05, sternum 0.30 long, 0.30 wide, coxae IV separated by 1.5 times their width. Posterior median eyes separated by less than half their diameter. Macrosetae as in male. Metatarsal trichobothria: Tm I: 0.32; Tm II: 0.38; Tm III: 0.29. Leg measurements: I 1.09 (0.32, 0.20, 0.24, 0.25, 0.18); II 1.02 (0.25, 0.19, 0.23, 0.20, 0.15); III 0.75 (0.19, 0.11, 0.13, 0.18, 0.14); IV 0.91 (0.25, 0.13, 0.21, 0.18, 0.14).

### Genus *Ogulnius* O. Pickard-Cambridge, 1882

*Ogulnius* O. Pickard-Cambridge, 1882: 432. Type species *Ogulnius obtectus* O. Pickard-Cambridge, 1882.

#### Ogulnius hapalus sp. n.

urn:lsid:zoobank.org:act:2CE2BCE0-15BC-488A-A363-872B6BA232F0

http://species-id.net/wiki/Ogulnius_hapalus

**Figure 19.**
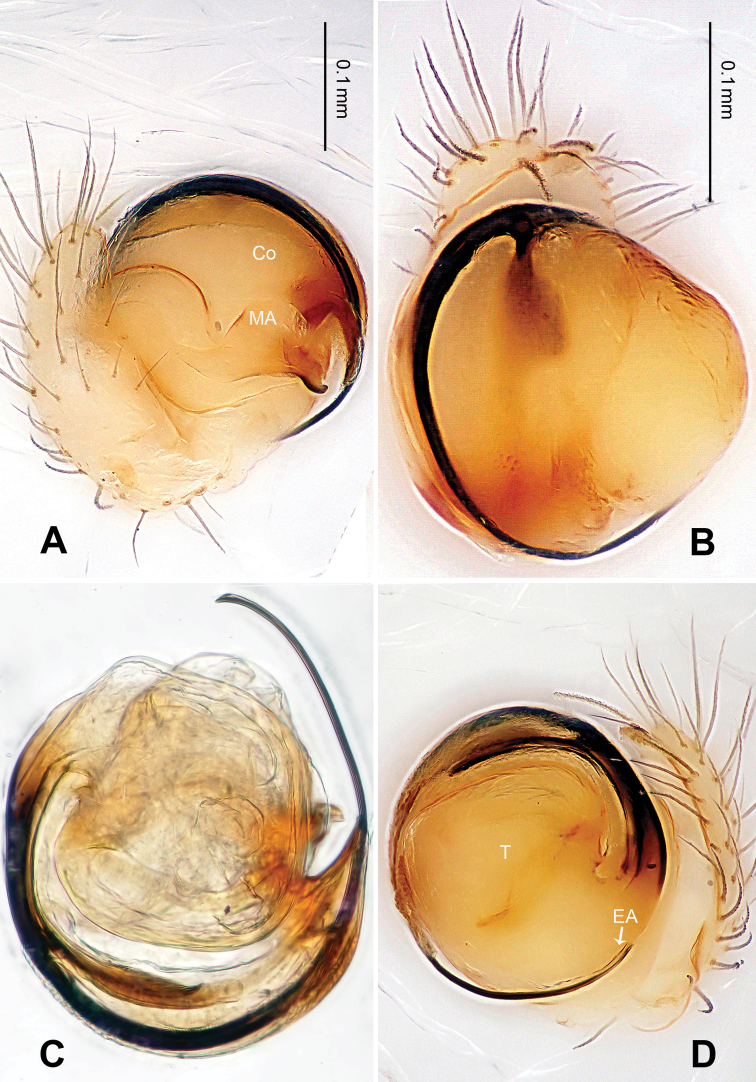
*Ogulnius hapalus* sp. n., male holotype. **A** Pedipalp, prolateral view **B** Pedipalp, ventral view **C** Embolic division, retrolateral view **D** Pedipalp, retrolateral view. **Co** conductor; **EA** embolic apophysis; **MA** median apophysis **T** tegulum. Scale bars: **D** as **A, C** as **B**.

Figs 19 [Fig F20] 21

##### Material examined.

Holotype: CHINA, Yunnan: Menglun Town: Xishuangbanna Botanical Garden, 21°55.035'N, 101°16.500'E, elevation ca 558 m, 22 July 2007, primary tropical seasonal rain forest, fogging, G. Zheng (IZCAS), 1 male.

Paratypes: [same data as holotype] (IZCAS), 2 males, 6 females.

##### Etymology.

This specific name describes the softness and fragility of this species. The Latin origin is ‘hapalus’ meaning ‘delicate and tender’; adjective.

##### Diagnosis.

**Figure 20. F20:**
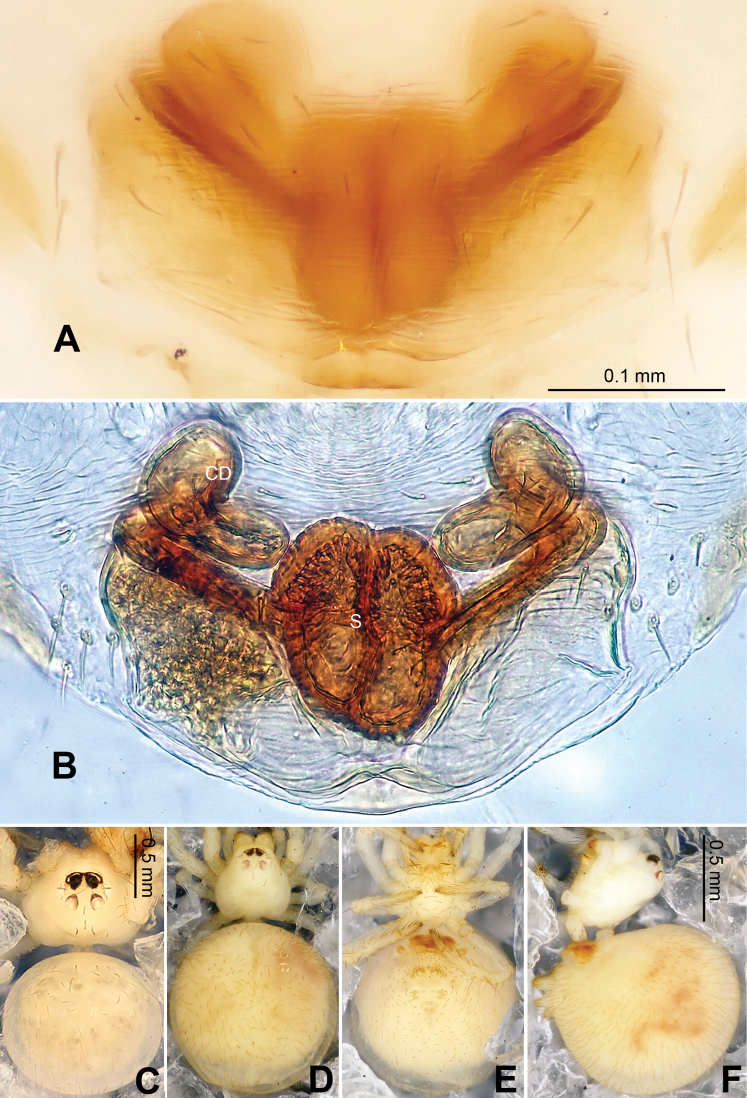
*Ogulnius hapalus* sp. n., male holotype (**C**) and female paratype (**A–B, D–F**). **A** Epigyne, ventral view **B** Vulva, dorsal view **C** Male habitus, dorsal view **D** Female habitus, dorsal view **E** Female habitus, ventral view **F** Female habitus, lateral view. **CD** copulatory duct; **S** spermatheca. Scale bars: **B** as **A, D, E** as **F**.

Though lack of one genetic feature, which is fourth legs are longer than the first legs (subequal in females), other generic characteristics of *Ogulnius* can be seen in this species: tapering whip-like embolic apophysis in males (Fig. 19C), transverse grooves on epigyne in females, separated and juxtaposed posterior median eyes (Coddington 1986). Males distinguished from other described *Ogulnius* species by the shape of median apophysis and the proportion of embolic apophysis. The median apophysis in *Ogulnius hapalus*.is similar to that in *Ogulnius gloriae* (Coddington 1986: fig. 99): mesally wide, with a projection oriented distoventrally (Fig. 19A). The embolic apophysis is proportionately longer than that in *Ogulnius barbandrewsi* (Miller et al. 2009: fig. 5F). The Females distinguished by the routing of copulatory ducts and the peanut-shaped spermathecae. The fold made by the copulatory ducts is bending outwardly (Fig. 20B), instead of inwardly as in *Ogulnius barbandrewsi* (Miller et al. 1986: fig. 3D). The posterior lip of epigyne in *Ogulnius hapalus* concave in stead of convex as in *Ogulnius pullus* (Brignoli 1981: fig.1).

##### Description.

Carapace pale yellow. Sternum ivory. Legs pale yellow, semi-transparent. Abdomen pale with soft, translucent cuticle.

**Figure 21.**
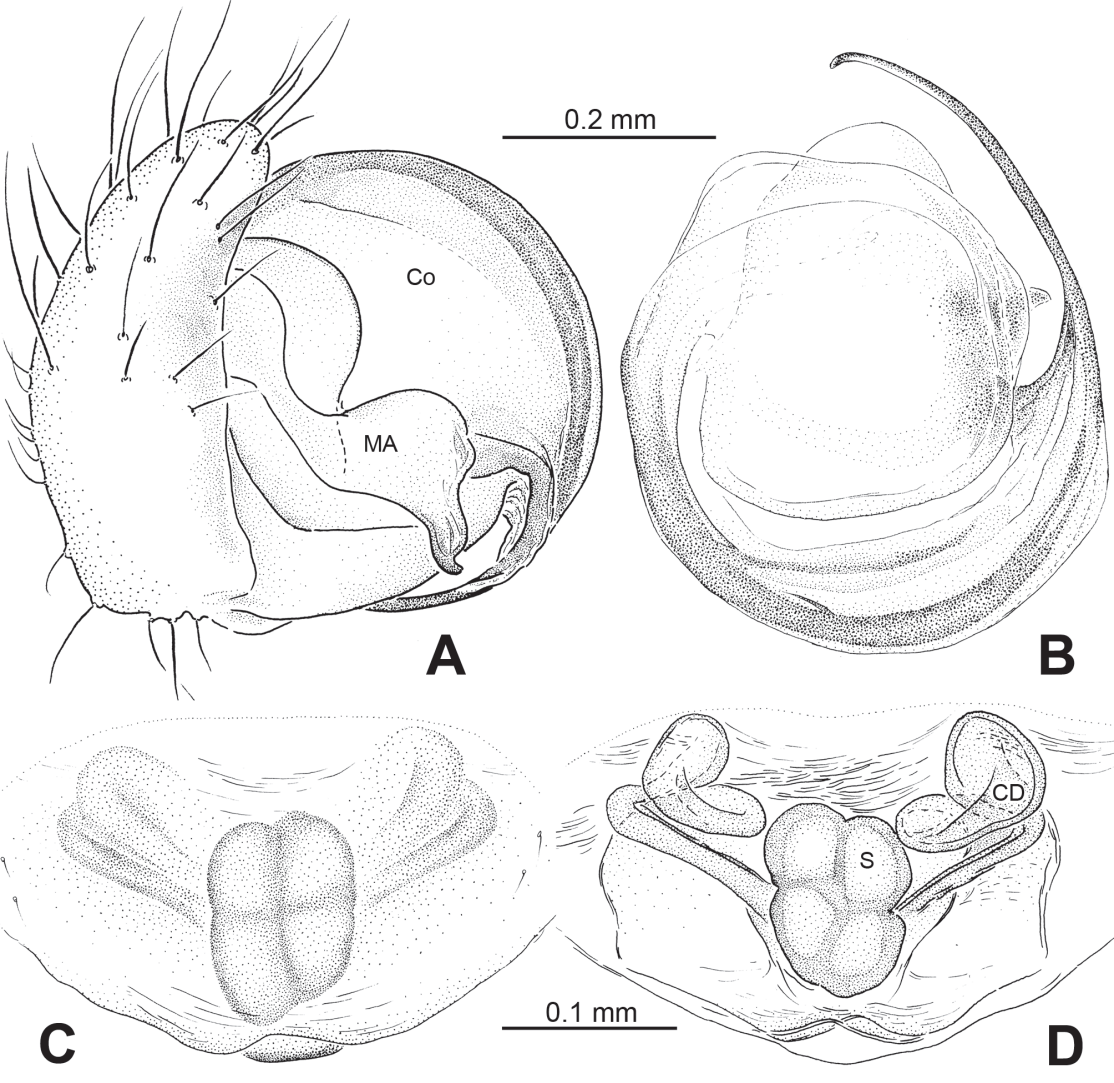
*Ogulnius hapalus* sp. n., male holotype (**A–B**) and female paratype (**C–D**). **A** Pedipalp, prolateral view **B** Embolic division, retrolateral view **C** Epigyne, ventral view **D** Vulva, dorsal view. **CD** copulatory duct; **Co** conductor; **MA** median apophysis; **S** spermatheca.

Male pedipalp: Patella with strong sinuous macroseta. Tibia with one trichobothrium. Paracymbium slim with filiform projection distally. Median apophysis curvy, mesally wide, with apex oriented distoventrally (Figs 19A, 21A). Conductor a translucent theca covering about two thirds length of the long filiform embolic apophysis (Figs 19C–D).

Vulva: Epigyne with transverse groove and a concave posterior margin (Fig. 20A). Spermathecae peanut-shaped, juxtaposed (Fig. 20B). Copulatory ducts bend downwardly at top and wide at entrance (Figs 20B, 21B).

Male: Total length 1.00, carapace 0.50 long, 0.40 wide, clypeus 0.09, sternum 0.25 long, 0.25 wide, coxae IV separated by 2.00 times their width. Posterior median eyes separated by 1.5 times their diameter. Macrosetae: Leg I: patella d 1, tibia d 1, p 1; Leg II: patella d 1, tibia d 1; Leg III: patella d 1, tibia d 1; Leg IV: patella d 1, tibia d 1. Metatarsal trichobothria: Tm I: 0.23; Tm II: 0.19; Tm III: 0.27. Leg measurements: I 1.00 (0.30, 0.18, 0.20, 0.20, 0.12); II 0.87 (0.26, 0.12, 0.20, 0.16, 0.13); III 0.57 (0.11, 0.10, 0.11, 0.15, 0.10); IV 0.64 (0.15, 0.10, 0.14, 0.15, 0.10).

Female: Total length 1.41, carapace: 0.50 long, 0.44 wide, clypeus 0.23, sternum 0.30 long, 0.25 wide, coxae IV separated by 2.00 times their width. Posterior median eyes separated 1.5 times their diameter. Macrosetae as in male. Metatarsal trichobothria: Tm I: 0.25; Tm II: 0.21; Tm III: 0.27. Leg measurements: I 0.95 (0.20, 0.25, 0.15, 0.20, 0.15); II 0.82 (0.25, 0.12, 0.15, 0.15, 0.15); III 0.62 (0.12, 0.10, 0.15, 0.15, 0.10); IV 0.90 (0.30, 0.15, 0.15, 0.20, 0.10).

### Genus *Theridiosoma* O. Pickard-Cambridge, 1879

*Theridiosoma* O. Pickard-Cambridge, 1879: 193. Type species *Theridiosoma argenteolum* O. Pickard-Cambridge, 1879 (= *Theridiosoma gemmosum* (L. Koch, 1878)).

#### Theridiosoma plumaria sp. n.

urn:lsid:zoobank.org:act:34DDB1DE-B435-4A41-8EF7-A40B43ACC9E0

http://species-id.net/wiki/Theridiosoma_plumaria

Figs 22 23

##### Material examined.

Holotype: CHINA, Hainan: Mt. Jianfengling National Nature Reserve: Tianchi watershed 18°44.383'N, 108°51.062'E, elevation ca 888 m, 19 July 2007, S. Li (IZCAS), 1 male.

Paratype: CHINA: Hainan: Mt. Bawangling National Nature Reserve, 5 kilometers past Dongerjianchazhan, 19°05.186'N, 109°11.802'E, elevation ca 1010 m, 25 July 2007, S. Li (IZCAS), 1 male.

##### Etymology.

**Figure 22.**
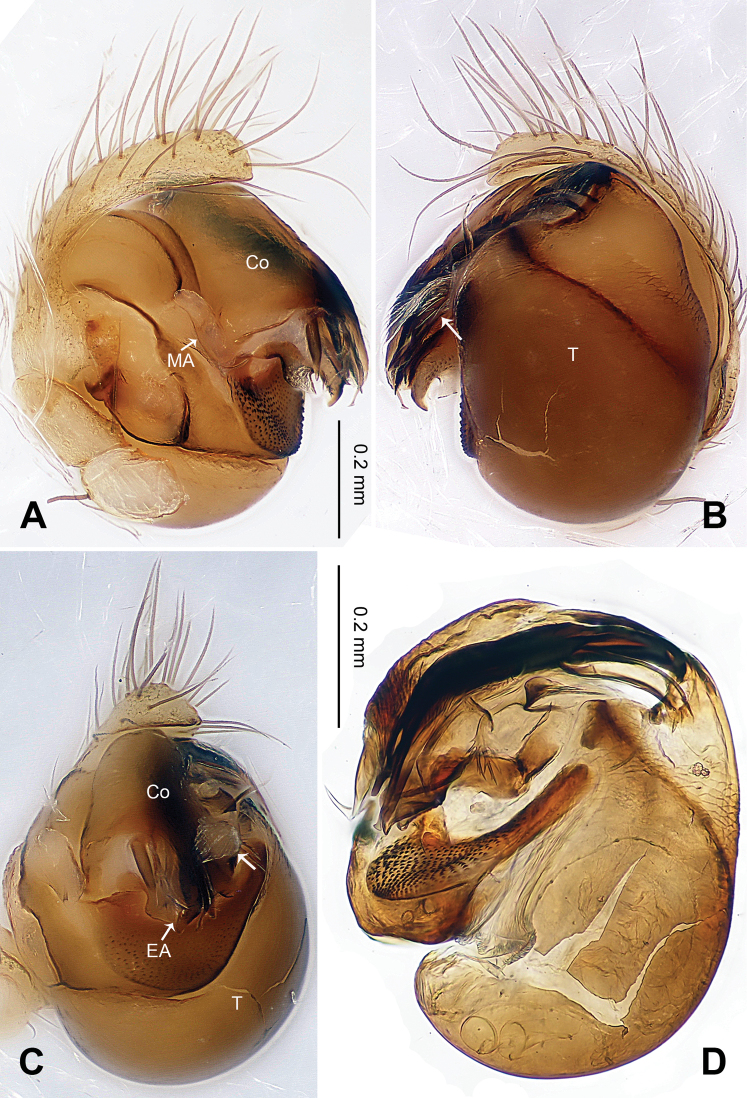
*Theridiosoma plumaria* sp. n., male holotype. **A** Pedipalp, prolateral view **B** Pedipalp, retrolateral view **C** Pedipalp, ventral view **D** Embolic division, retrolateral view. **Co** conductor; **EA** embolic apophysis; MA median apophysis; **T** tegulum. Scale bars: **B, C** as **A**.

The specific name was taken from the Latin word ‘plumarius’ which means ‘of feathers’. It refers to the plumose branching of its conductor theca (Figs 22B–C); adjective.

##### Diagnosis.

Males, similar to *Theridiosoma gemmosum* (L. Koch) (Coddington 1986: figs 134, 135), distinguished from any other known male *Theridiosoma* from Asia by the following characters: broader, slightly groovy median apophysis with an acuminated tip, and the exposed branchings of embolic apophysis (Fig. 22A).

##### Description.

Carapace yellow tan. Sternum yellow with brown margins. Legs yellow. Abdomen tan with sparse silver specks and symmetric dark grey patches.

**Figure 23.**
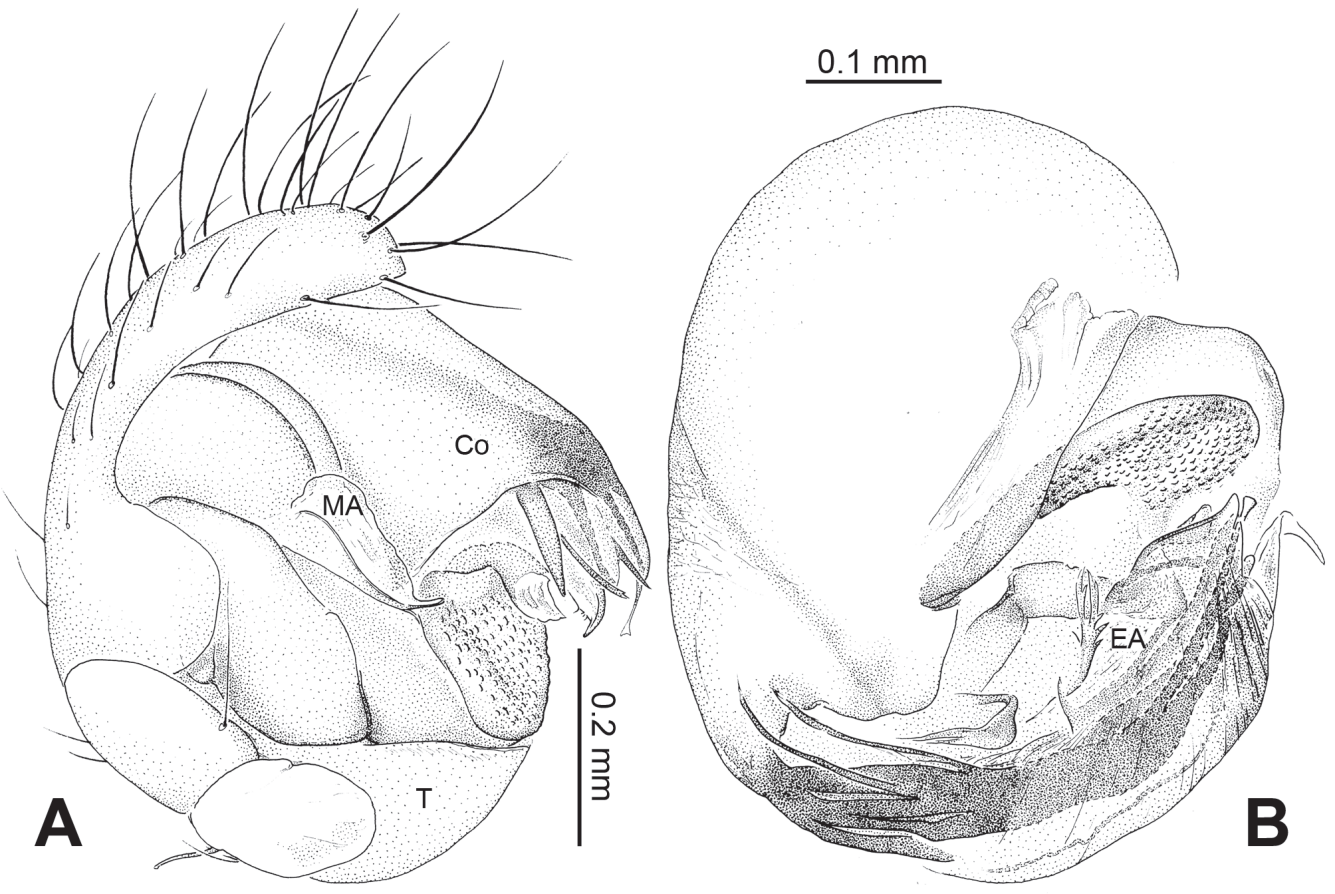
*Theridiosoma plumaria* sp. n., male holotype. **A** Pedipalp, prolateral view **B** Embolic division, retrolateral view. **Co** conductor; **EA** embolic apophysis; **MA** median apophysis; **T** tegulum.

Male pedipalp: Patella with strong macroseta. Tibia with two trichobothria. Paracymbium hooked with pointed distal end. Tegulum large with tuberculate mesal lobe (Figs 22A, D). Median apophysis a curved lobe attenuate distally. Conductor a translucent theca covering fragmented embolic apophysis, with plumose branching and erect tip near tegulum (Fig. 22B). Embolic apophysis fragment tips protruding out of the conductor (Figs 22A–C). Embolic apophysis fragments long, slim (Figs 22D, 23B).

Male: Total length 1.60, carapace 0.80 long, 0.80 wide, clypeus 0.20, sternum 0.40 long, 0.40 wide, coxae IV separated by their width. Posterior median eyes separated by less than half their diameter. Macrosetae: Leg I: femur p 1, patella d 1, tibia d 1, p 1, r 1; Leg II: femur r 1, patella d 1, tibia d 1, p 1, r 1; Leg III: patella d 1, tibia p 1, r 1, metatarsus p 1; Leg IV: patella d 2, tibia d 1. Metatarsal trichobothria: Tm I: 0.0.25; Tm II: 0.19. Leg measurements: I 3.16 (0.93, 0.35, 0.75, 0.75, 0.38); II 2.80 (0.88, 0.28, 0.63, 0.63, 0.38); III 1.66 (0.50, 0.18, 0.35, 0.38, 0.25); IV 1.83 (0.50, 0.25, 0.38, 0.45, 0.25).

Female unknown

#### Theridiosoma triumphalis sp. n.

urn:lsid:zoobank.org:act:958EEF84-677E-4E46-B673-AA78B5FF63E6

http://species-id.net/wiki/Theridiosoma_triumphalis

Figs 24 25

##### Material examined.

Holotype: CHINA, Hainan: Mt. Jianfengling National Nature Reserve: Tianchi watershed, 18°44.383'N, 108°51.062'E, elevation ca 888 m, 19 July 2007, S. Li (IZCAS), 1 male.

##### Etymology.

**Figure 24.**
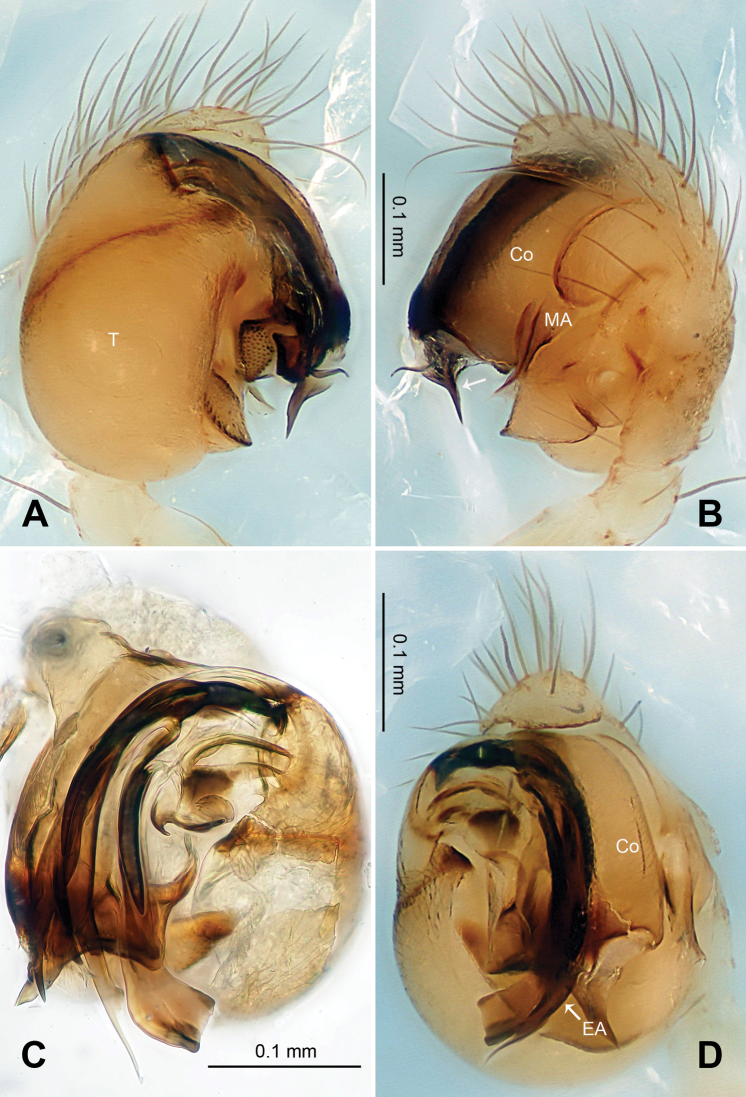
*Theridiosoma triumphalis* sp. n., male holotype. **A** Right pedipalp, prolateral view **B** Right pedipalp, retrolateral view **C** Embolic division, retrolateral view **D** Right pedipalp, ventral view. **Co** conductor; **EA** embolic apophysis; **MA** median apophysis; **T** tegulum. Scale bars: **B** as **A**.

**Figure 25.**
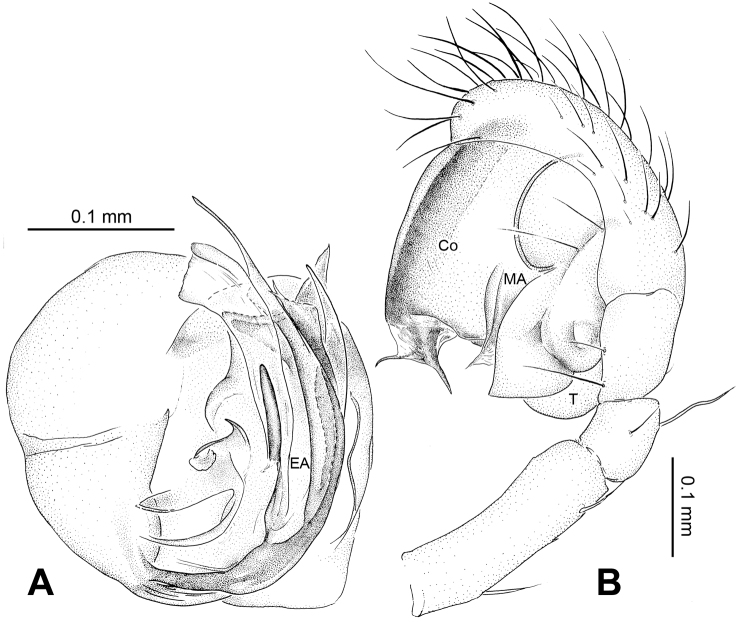
*Theridiosoma triumphalis* sp. n., male holotype. **A** Embolic division, retrolateral view **B** Right pedipalp, prolateral view. **Co** conductor; **EA** embolic apophysis; **MA** median apophysis; **T** tegulum.

The specific Latin word ‘triumphalis’ meaning ‘of victory’ refers to the 'V'-shaped conformation made by the two embolic apophysis fragments (Figs 24A, B, 25B); adjective.

##### Diagnosis.

Males distinguished from other described Asian *Theridiosoma* species by the shape of median apophysis: a small, curved projection with an attenuated tip extending distoventrally (Fig. 24B). Embolic apophysis fragmented, similar to *Theridiosoma caaguara* (Rodrigues and Ott 2005: figs 4, 5), but different in details: four pieces of different shapes, the longest one with a whip-like tip, the longer one broad, with a blunt end (Fig. 24C).

##### Description.

Carapace yellow tan. Sternum yellow with dark margins. Legs yellow. Due to poor preservation condition, abdomen too shriveled to make out its original color pattern.

Male pedipalp: Patella with strong macroseta. Paracymbium elongate with attenuated tip. Tegulum smooth with tuberculate ventral ridge. Median apophysis curved lobe with attenuated distal end. Conductor translucent theca covering most fragmented embolic apophysis, with a small rough ventral area (Fig. 24A). Two embolic apophysis fragment tips protruding out of the conductor tip to form a 'V'-shaped conformation (Fig. 24B). Embolic apophysis with four separate sclerotic fragments (Figs 24C, 25A).

Male: Total length 1.7, carapace 0.50 long, 0.50 wide, clypeus 0.09, sternum 0.31 long, 0.31 wide, coxae IV separated by their width. Posterior median eyes separated by less than half their diameter. Macrosetae: Leg I: femur r 1, patella d 1, tibia d 1, p 1; Leg II: patella d 1, tibia d 2, p 1; Leg III: tibia p 1; Leg IV: tibia d 1. Metatarsal trichobothria: Tm I: 0.21; Tm II: 0.27; Tm III: 0.20. Leg measurements: I 2.01 (0.63, 0.25, 0.50, 0.38, 0.25); II 1.76 (0.50, 0.25, 0.38, 0.38, 0.25); III 1.33 (0.30, 0.13, 0.15, 0.25, 0.15); IV 1.14 (0.38, 0.13, 0.25, 0.25, 0.13).

Female unknown

#### Theridiosoma vimineum sp. n.

urn:lsid:zoobank.org:act:7E3616F4-8E17-4E5D-9892-98A2860B8AC3

http://species-id.net/wiki/Theridiosoma_vimineum

Figs 26 27

##### Material examined.

Holotype: CHINA, Yunnan: Menglun Town: Xingshuangbanna Botanical Garden, 21°55.428'N, 101°16.441'E, elevation ca 598 m, 19–26 May 2007, primary tropical seasonal rain forest, searching, G. Zheng (IZCAS), 1 male.

##### Etymology.

**Figure 26.**
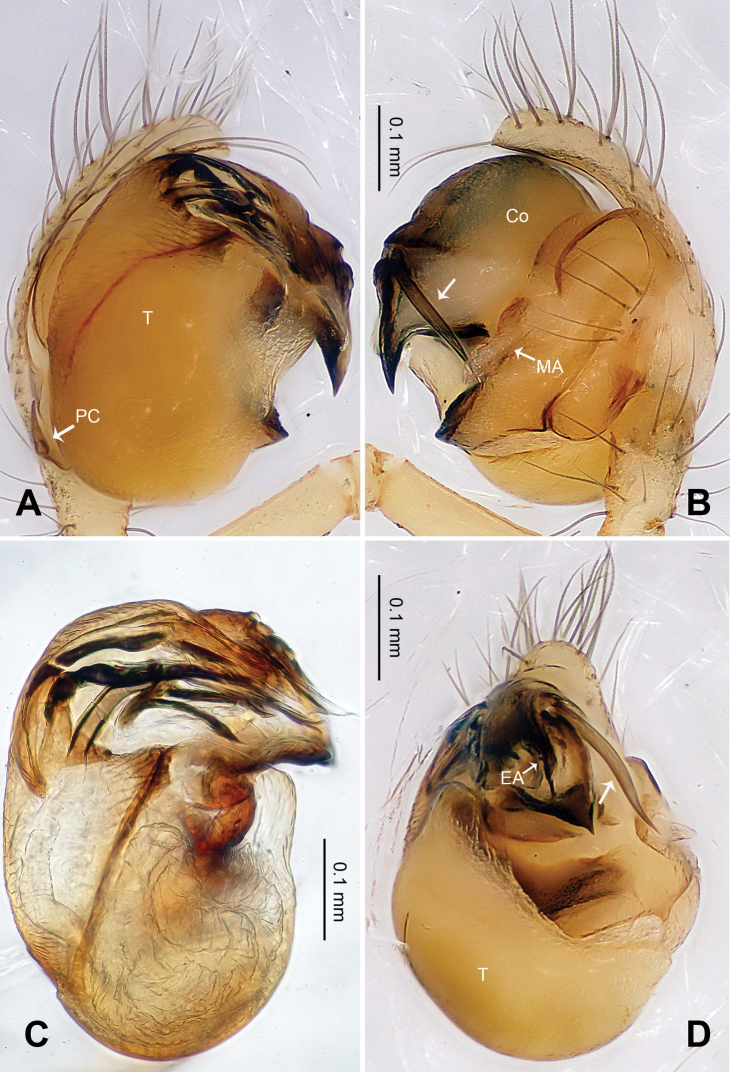
*Theridiosoma vimineum* sp. n., male holotype. **A** Right pedipalp, prolateral view **B** Right pedipalp, retrolateral view **C** Embolic division of right pedipalp, retrolateral view **D** Right pedipalp, ventral view. **Co** conductor; **EA** embolic apophysis; **MA** median apophysis; **PC** paracymbium; **T** tegulum. Scale bars: **A** as **B**.

The specific name is derived from Latin word ‘vimineus’ meaning ‘pliant’, refers to the long, pliant apophysis that protrudes from the conductor (Fig. 26B); adjective.

##### Diagnosis.

Males similar to *Theridiosoma semiargentum* (Simon) (Coddington 1986: figs 154, 156), but different in the details of embolic apophysis fragments and the shape of median apophysis. The apophysis protruding from the conductor is longer and slimmer in *Theridiosoma vimineum*, spur on the conductor is absent, and the median apophysis lacks a pointed hook at the tip.

##### Description.

Carapace yellow. Sternum tan with dark margins. Legs yellow. Abdomen tan with light grey spots randomly distributed on the dorsal area.

**Figure 27.**
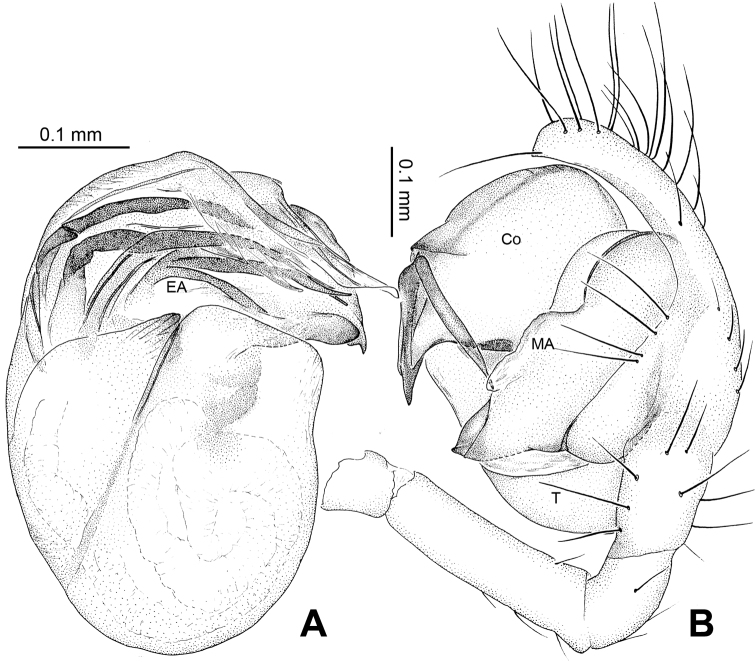
*Theridiosoma vimineum* sp. n., male holotype. **A** Embolic division of right pedipalp, retrolateral view **B** Right pedipalp, prolateral view. **Co** conductor; **EA** embolic apophysis; **MA** median apophysis; **T** tegulum.

Male pedipalp: Patella with macroseta. Tibia with one trichobothrium. Paracymbium elongate with sharp tip. Tegulum smooth with rough mesal lobe. Median apophysis slightly grooved, semi-transparent at the distal end (Figs 26B, 27B). Conductor translucent theca with a hooked end oriented proximally (Fig. 26A). A piece of long, flat embolic apophysis fragment with a attenuated tip protruding from the ridge of conductor and stretching toward median apophysis (Figs 26B, D). Embolic apophysis fragmented into long, slim bristles (Fig. 26C).

Male: Total length 1.75, carapace 0.95 long, 0.50 wide, clypeus 0.19, sternum 1.54 long, 0.35 wide, coxae IV separated by their width. Posterior median eyes separated by their diameter. Macrosetae: Leg I: femur d 1, patella d 2, tibia d 2, p 1; Leg II: patella d 2, tibia d 2, r 1; Leg III: patella d 1, tibia r 2; Leg IV: tibia d 1. Metatarsal trichobothria: Tm I: 0.23; Tm II: 0.20. Leg measurements: I 2.18 (0.68, 0.25, 0.50, 0.50, 0.25); II 1.86 (0.63, 0.20, 0.38, 0.40, 0.25); III 1.03 (0.35, 0.13, 0.15, 0.25, 0.15); IV 1.31 (0.38, 0.18, 0.30, 0.30, 0.15).

Female unknown

### Genus *Zoma* Saaristo, 1996

*Zoma* Saaristo, 1996: 51. Type species: *Zoma zoma* Saaristo, 1996.

#### Zoma fascia sp. n.

urn:lsid:zoobank.org:act:4FC78696-B0B2-413F-B8F1-A4FF9D25591F

http://species-id.net/wiki/Zoma_fascia

**Figure 28.**
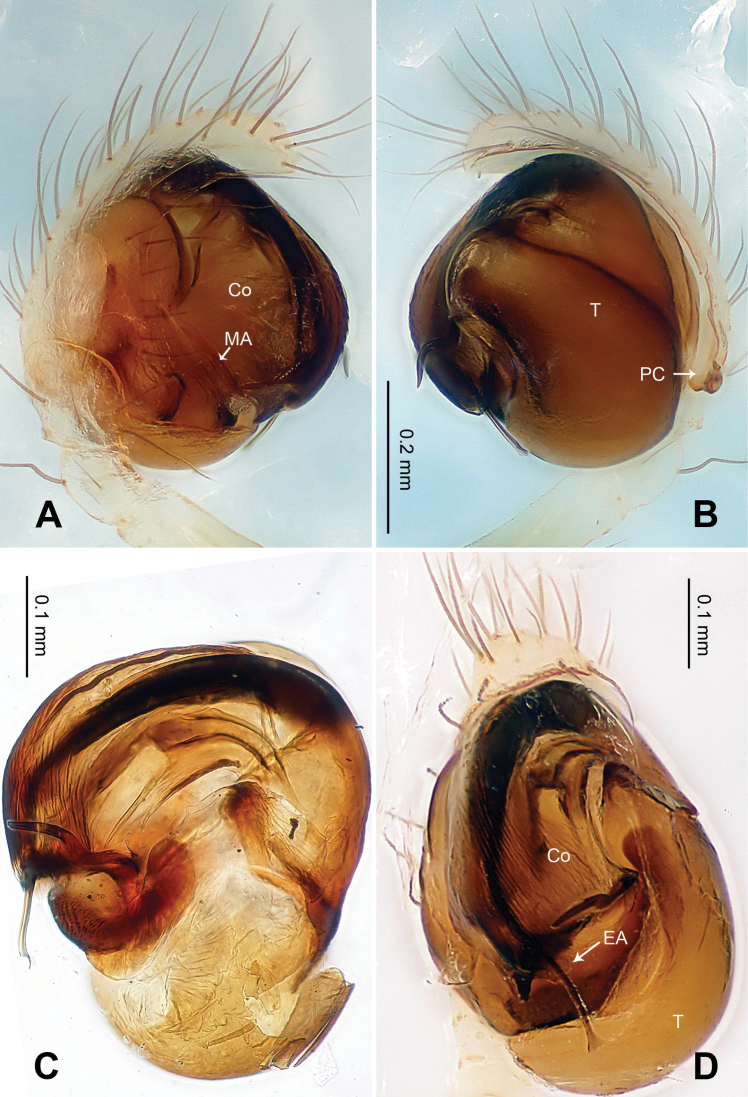
*Zoma fascia* sp. n., male holotype. **A** Pedipalp, prolateral view **B** Pedipalp, retrolateral view **C **Embolic division, retrolateral view **D** Pedipalp, ventral view. **Co** conductor; **EA** embolic apophysis; **MA** median apophysis; **PC** paracymbium; **T** tegulum. Scale bars: **A** as **B**.

Figs 28 [Fig F29] 30

##### Material examined.

Holotype: CHINA: Hainan: Mt. Bawangling National Nature Reserve, 5 kilometers past Dongerjianchazhan, 19°05.186'N, 109°11.802'E, elevation ca 1010 m, 25 July 2007, S. Li (IZCAS), 1 male.

Paratypes: CHINA: Hainan: Mt. Diaoluoshan National Nature Reserve, Diaoluoshan Resort, 18°43.766'N, 109°51.815'E, elevation ca 1010 m, 15 August 2007, S. Li (IZCAS), 1 female; Mt. Jianfengling National Nature Reserve, Huxiaolongyin scenery, on the mountain by the river, 18°45.159'N, 108°54.604'E, elevation ca 900 m, 20 July 2007, S. Li (IZCAS), 1 female; Mt. Limushan Provincial Nature Reserve, Qulinggulinyuan, 19°10.686'N, 109°44.490'E, elevation ca 654 m, 12 August 2007, C.X. Wang (IZCAS), 1 female; [same data as holotype] (IZCAS), 1 female; Mt. Jianfengling National Nature Reserve, east valley of Tianchi, 18°54.691'N, 108°51.588'E, elevation ca 811 m, 17 July 2007, S. Li (IZCAS), 1 female.

##### Etymology.

The specific name comes from a Latin word ‘fascia’, which refers to the silver band on its abdomen; noun.

##### Diagnosis.

Males distinguished by the presence of a filiform embolic apophysis extending beyond the conductor tip and a 'Z'-shaped embolus (Figs 28B, D). Exposed portion of embolic apophysis is smaller compared to *Zoma didaiyin* (Miller et al. 2009: fig. 10F).

**Figure 29. F29:**
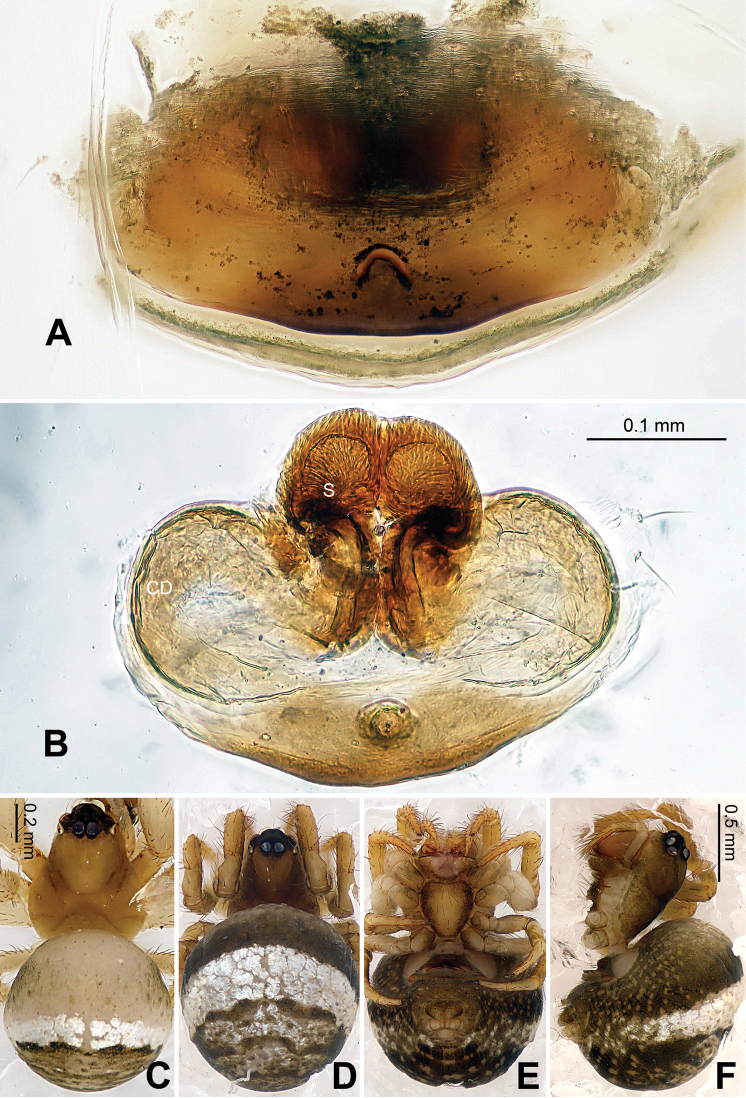
*Zoma fascia* sp. n., male holotype (**C**) and female paratype (**A–B, D–F**). **A** Epigyne, ventral view **B** Vulva, dorsal view **C** Male habitus, dorsal view **D** Female habitus, dorsal view **E** Female habitus, ventral view **F** Female habitus, lateral view. **CD** copulatory duct; **S** spermatheca. Scale bars: **A** as **B, D, E** as **F**.

Female distinguished from *Zoma didaiyin* by the mildly convex posterior margin of the epigyne and the higher position of the spermathecae relative to the copulatory ducts (Figs 29A, B).

##### Description.

Carapace brownish yellow in males, brown in females. Sternum yellow with dark brown margins. Legs yellow. Abdomen beige in males, dark grey in females with silver pecks forming a transverse belt.

Male pedipalp: Patella with macroseta. Tibia with one trichobothrium. Paracymbium hooked with short, pointed tip (Fig. 28B). Median apophysis lightly sclerotized, similar to *Zoma didaiyin* (Miller et al. 2009: fig. 10D). Conductor translucent. Embolic apophysis filiform, with triangular tip (Fig. 28C).

Vulva: Epigyne a flat orange plate with a low median pit. Spermathecae subspherical, juxtaposed. Copulatory ducts follow simple curve (Fig. 29A, B) .

Male: Total length 1.52, carapace 0.72 long, 0.60 wide, clypeus 0.14, sternum 0.37 long, 0.31 wide, coxae IV separated by their width. Posterior median eyes separated by less than half their diameter. Macrosetae: Leg I: femur p 1, patella d 1, tibia d 1, p 1, r 1; Leg II: patella d 2, tibia d 3, v 2, r 1; Leg III: patella d 1, tibia d 1; Leg IV: patella d, tibia d 1. Metatarsal trichobothria: Tm I: 0.21; Tm II: 0.20; Tm III: 0.27. Leg measurements: I 2.19 (0.70, 0.25, 0.50, 0.47, 0.27); II 1.99 (0.63, 0.23, 0.44, 0.39, 0.30); III 1.16 (0.31, 0.23, 0.22, 0.23, 0.17); IV 1.45 (0.39, 0.21, 0.31, 0.31, 0.23).

Female: Total length 1.80, carapace 0.88 long, 1.00 wide, clypeus 0.18, sternum 0.50 long, 0.30 wide, coxae IV separated by their width. Posterior median eyes separated by less than half their diameter. Macrosetae as in male. Metatarsal trichobothria: Tm I: 0.30; Tm II: 0.19; Tm III: 0.27. Leg measurements: I 2.11 (0.63, 0.31, 0.47, 0.39, 0.31); II 1.97 (0.63, 0.31, 0.39, 0.39, 0.25); III 1.47 (0.47, 0.23, 0.31, 0.25, 0.21); IV 1.16 (0.31, 0.23, 0.23, 0.23, 0.16).

**Figure 30.**
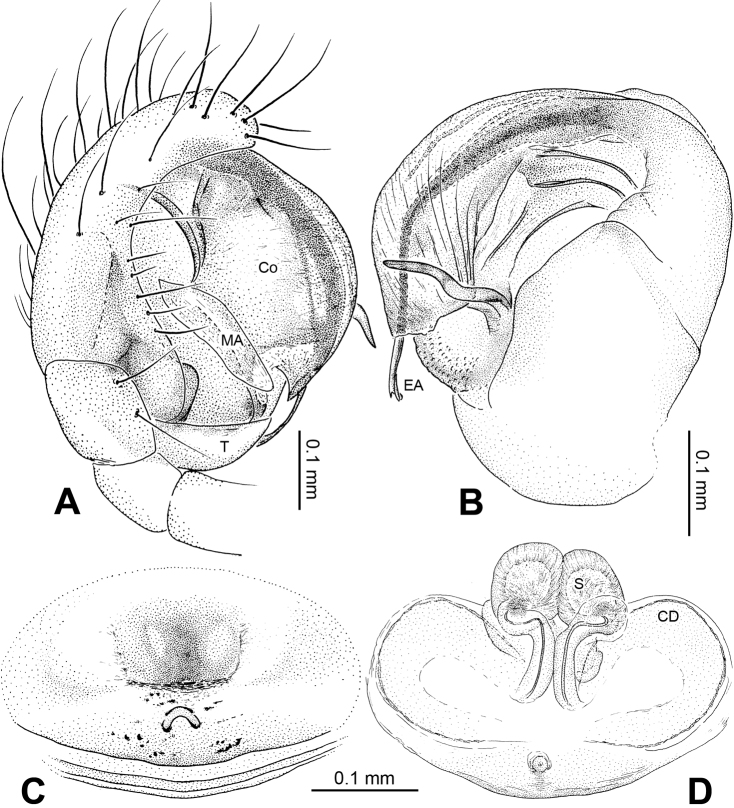
*Zoma fascia* sp. n., male holotype (**A–B**) and paratype female (**C–D**). **A** Pedipalp, prolateral view **B** Embolic division, retrolateral view **C** Epigyne, ventral view **D** Vulva, dorsal view. **CD** copulatory duct; **Co** conductor; **EA** embolic apophysis; **MA** median apophysis; **S** spermatheca. **T** tegulum.

**Figure 31.**
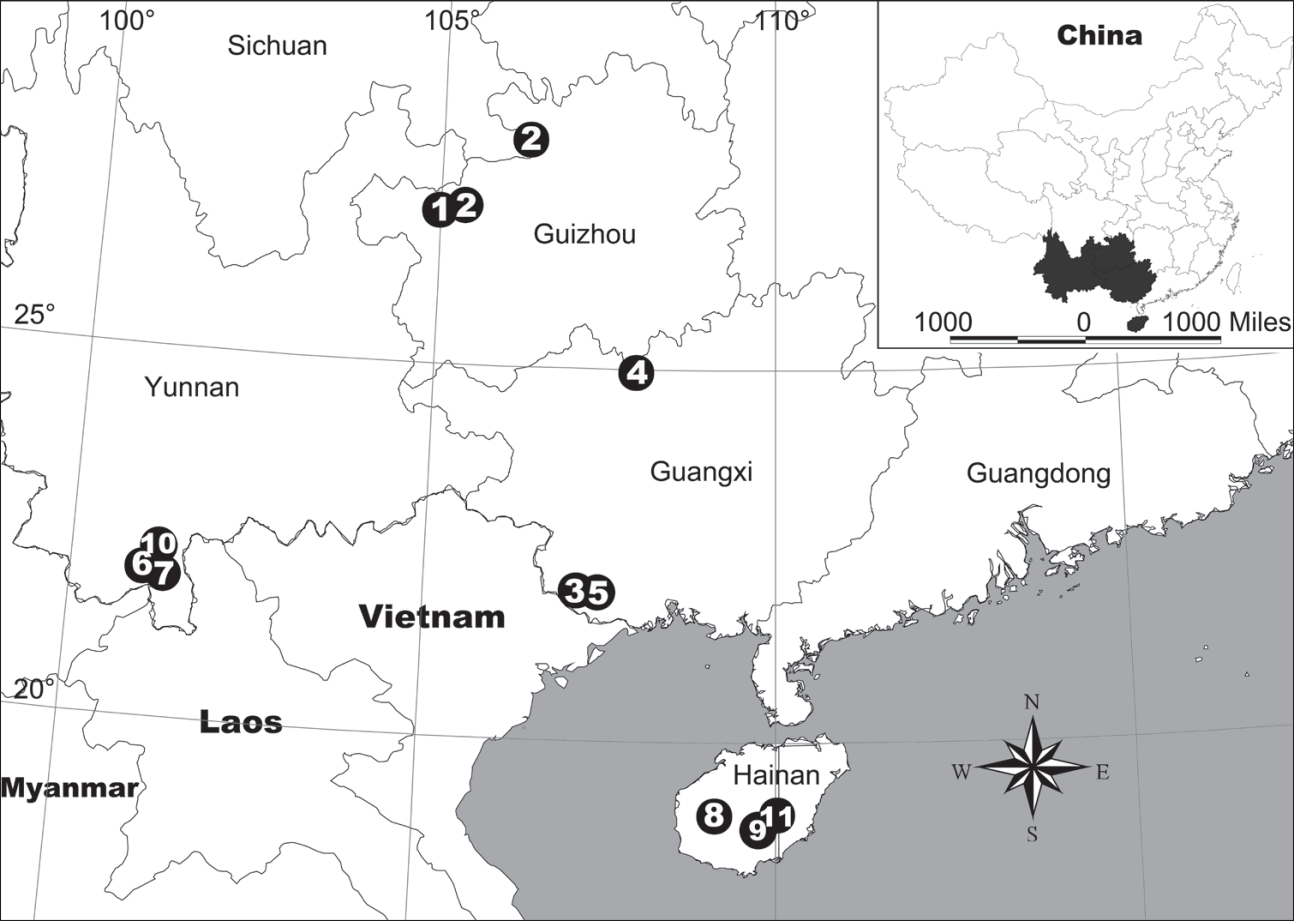
Locality records for eleven theridiosomatid spider species in China. **1** *Alaria chengguanensis* gen. n., sp. n. **2** *Baalzebub rastrarius* sp. n. **3** *Baalzebub youyiensis* sp. n. **4** *Karstia nitida* sp. n. **5** *Karstia prolata* sp. n. **6** *Menglunia inaffecta* gen. n., sp. n. **7** *Ogulnius hapalus* sp. n. **8** *Theridiosoma plumaria* sp. n. **9** *Theridiosoma triumphalis* sp. n. **10** *Theridiosoma vimineum* sp. n. **11** *Zoma fascia* sp. n.

## Supplementary Material

XML Treatment for Alaria

XML Treatment for Alaria chengguanensis

XML Treatment for Baalzebub rastrarius

XML Treatment for Baalzebub youyiensis

XML Treatment for Karstia nitida

XML Treatment for Karstia prolata

XML Treatment for Menglunia

XML Treatment for Menglunia inaffecta

XML Treatment for Ogulnius hapalus

XML Treatment for Theridiosoma plumaria

XML Treatment for Theridiosoma triumphalis

XML Treatment for Theridiosoma vimineum

XML Treatment for Zoma fascia
